# Effects of Oligolysine-Polyethylene Glycol Coating on the Biodistribution of Wireframe DNA Origami Nanosheets in Zebrafish Embryos

**DOI:** 10.1021/acsnano.5c05801

**Published:** 2025-09-03

**Authors:** Christina Kolonelou, Enya Engström, Lars Bräutigam, Steven Edwards, José M. Dias, Joel Spratt, Christos Karampelias, Iris Rocamonde-Lago, Björn Högberg, Stefan Wennmalm, Hjalmar Brismar, Olov Andersson, Ana I. Teixeira

**Affiliations:** 1 Department of Physiology and Pharmacology, 27106 Karolinska Institutet, Stockholm 171 77, Sweden; 2 Department of Comparative Medicine, Karolinska Institutet, Stockholm 171 77, Sweden; 3 Science for Life Laboratory, Department of Applied Physics, 7655 KTH Royal Institute of Technology, Solna 171 21, Sweden; 4 Department of Cell and Molecular Biology, Karolinska Institutet, Stockholm 171 77, Sweden; 5 Department of Medical Biochemistry and Biophysics, 27106 Karolinska Institutet, Stockholm 171 77, Sweden; 6 Department of Medical Cell Biology, Uppsala University, Uppsala 751 23, Sweden

**Keywords:** biodistribution, DNA origami, zebrafish, single-cell RNA sequencing, light sheet fluorescence microscopy

## Abstract

DNA origami-based nanotechnology is a versatile tool for exploring fundamental biological questions and holds significant promise for future biomedical applications. Here, we leverage the optical transparency of the embryonic zebrafish to analyze live embryos injected intravenously with fluorescently labeled wireframe DNA origami nanosheets. Our approach integrated long-term, high-resolution imaging of transgenic live zebrafish embryos with single-cell RNA sequencing to elucidate the effects of oligolysine-polyethylene glycol copolymer (K-PEG) coating on the biodistribution of fluorescence signal in embryos injected with wireframe DNA origami nanosheets. We observed rapid accumulation of fluorescence signal in the caudal hematopoietic tissue (CHT). K-PEG coating mitigated the accumulation of fluorescence signal in CHT, enabling increased detection of signal in other tissues. Our findings highlighted the pivotal role of scavenger endothelial cells in DNA origami clearance, with K-PEG enabling the prolonged detection of fluorescence signal at the CHT. Furthermore, using a transgenic zebrafish line designed for targeted macrophage ablation, we found that macrophages contribute to the clearance of fluorescence signal in embryos injected with the noncoated but not with K-PEG-coated nanosheets. This study introduces a framework for the analyses of the biodistribution and clearance of DNA origami nanostructures *in vivo* with single-cell resolution in zebrafish models.

## Graphical abstract

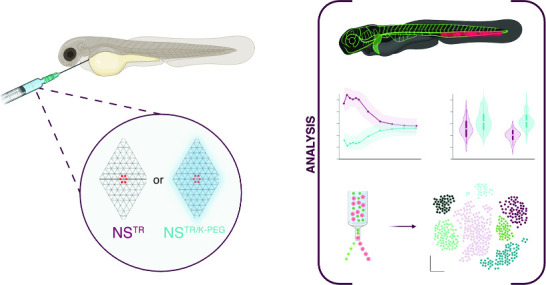

## Main

In recent years, there has been significant progress in the field of DNA nanotechnology toward enabling *in vivo* applications. Inherently biocompatible and biodegradable, DNA nanostructures are suitable for studies in animal models. Wireframe DNA architectures are particularly advantageous for *in vivo* studies as they are more stable under physiological conditions compared to conventional DNA origami, due to their lower packing density.^1–4^ Additionally, tailoring the number of DNA duplexes per edge in the polyhedral meshes enables control over the structural rigidity of the nanostructures. Single-duplex edges are the most flexible^2^ whereas double-duplex^4,5^ or six-helix bundle edges^6–8^ offer increased rigidity and shape fidelity. Apart from structural design, DNA origami can be further tailored through surface modifications and the incorporation of biomolecules to control their behavior *in vivo*. Several approaches have been explored to enhance the stability and circulation times of DNA nanostructures in mice, including coating the nanostructures with oligolysine-polyethylene glycol copolymers (K-PEG), which neutralize the negative charge and protect the nanostructures from DNase-mediated degradation.^9,10^ While the effects of K-PEG coatings on DNA nanostructures remain incompletely understood, variants of K-PEG copolymers have been shown to inhibit scavenger receptor-mediated uptake of nanoparticles in the liver.^11,12^ To enable tissue specific targeting, DNA origami nanostructures have been modified with antibodies, aptamers, or other bioactive ligands.^13–19^ These studies have demonstrated the feasibility of using DNA origami nanostructures for targeting specific tissues, such as the immune system or tumors. In contrast, DNA nanostructures without targeting moieties have been shown to accumulate primarily in the liver and kidney and have been proposed as a potential therapeutic strategy to mitigate kidney damage.^18,19^ In recent years, there has been a rapid increase in the number of studies investigating the therapeutic potential of DNA origami-based nanomedicines in mouse disease models.^9,16,20–28,29^ However, the biodistribution of fluorescently labeled DNA origami-based nanomedicines in mice is typically analyzed using *in vivo* imaging systems (IVIS)^9,21,22^ or positron emission tomography (PET),^18,30^ which have a resolution in the millimeter-scale, far from single cell resolution. Single-cell analyses methods have the potential to improve both resolution and sensitivity in the assessment of the interactions of DNA nanostructures with target cells, including their potential immunogenicity and toxicity.

Here we propose to use zebrafish embryo to study the distribution of DNA origami. The zebrafish (*Danio rerio*) embryo has emerged as a promising *in vivo* model in nanomedicine.^31^ Its optical transparency allows for real-time observation of intravenously administered fluorescently labeled objects in live animals. Therefore, intravital imaging of zebrafish embryos can provide whole-body information on nanoparticle targeting and clearance in live animals.^32–34^ In addition, zebrafish have a robust immune system, resembling that of mammals.^35–38^ However, innate immunity starts at embryogenesis in zebrafish, marked by the emergence of macrophages from the mesoderm,^39^ while adaptive immunity is activated at 3 weeks post fertilization.^40^ This temporal separation of the onset of the innate and adaptive immunity in zebrafish embryos can be leveraged in the study of the interactions of nanostructures with the immune system. We analyzed live zebrafish embryos injected with fluorescently labeled wireframe DNA nanosheets with or without K-PEG coating, using advanced imaging methods, including light sheet fluorescence microscopy (LSFM), Airyscan confocal microscopy, and Fluorescence Correlation Spectroscopy (FCS), coupled with single-cell RNA sequencing. Further, we explored the role of the innate immune system using targeted ablation of macrophages in a zebrafish model. Together, our results provide insights into the effects of K-PEG on the fate of wireframe DNA origami nanostructures in zebrafish embryos.

## Results

### Wireframe DNA Origami Nanosheets for *In Vivo* Assessment

We used wireframe scaffolded DNA origami to produce single layer two-dimensional sheets (Figure 1a, Figure S1a,b), herein referred to as NanoSheets (NS), which remain stable in physiological buffers, unlike DNA nanostructures constructed with tightly packed helices.^1,2^ To detect the NS, we designed six protruding ssDNA strands, centrally located on their surface, which hybridize to complementary ssDNA strands conjugated with the fluorophore Texas Red (TR), referred to as NS^TR^ (Figure 1a). Further, we employed a coating strategy widely used to enhance nanostructure stability,^27^ whereby positively charged oligolysine conjugated to polyethylene glycol (K-PEG) electrostatically interacts with the negatively charged DNA nanostructures, NS^TR/K‑PEG^ (Figure 1a). Imaging of NS structures folded in PBS using atomic force microscopy (AFM) and transmission electron microscopy (TEM) confirmed their self-assembly and integrity (Figure 1b, Figure S2a,b). Interestingly, TEM imaging revealed that the K-PEG coating was associated with a less well-defined nanosheet shape, and a more compacted appearance compared to the noncoated structures. Agarose gel electrophoresis analysis confirmed that the NanoSheets folded properly and were fluorescently labeled (Figure S2c). The number of Texas Red fluorophores per structure was estimated to be approximately three fluorophores, both for NS^TR^ and NS^TR/K‑PEG^, using a microplate fluorescence assay (Figure S2d). Further, agarose gel electrophoresis showed that NS^TR/K‑PEG^ had a neutral surface charge and were thereby retained in the well (Figure 1c). Removal of the K-PEG coating by incubation with chondroitin sulfate restored band migration, confirming that the coating did not cause irreversible conformational alterations of the NanoSheets (Figure S2e). We also investigated the stability of NS^TR^ and NS^TR/K‑PEG^ for 72 h at 28 °C in the presence of DNase I (Figure S3), confirming that the nanostructures were preserved (Figure S3a) and that fluorophores remained incorporated (Figure S3b) under *in vitro* conditions that mimic physiological conditions in zebrafish embryos.

**Figure 1:**
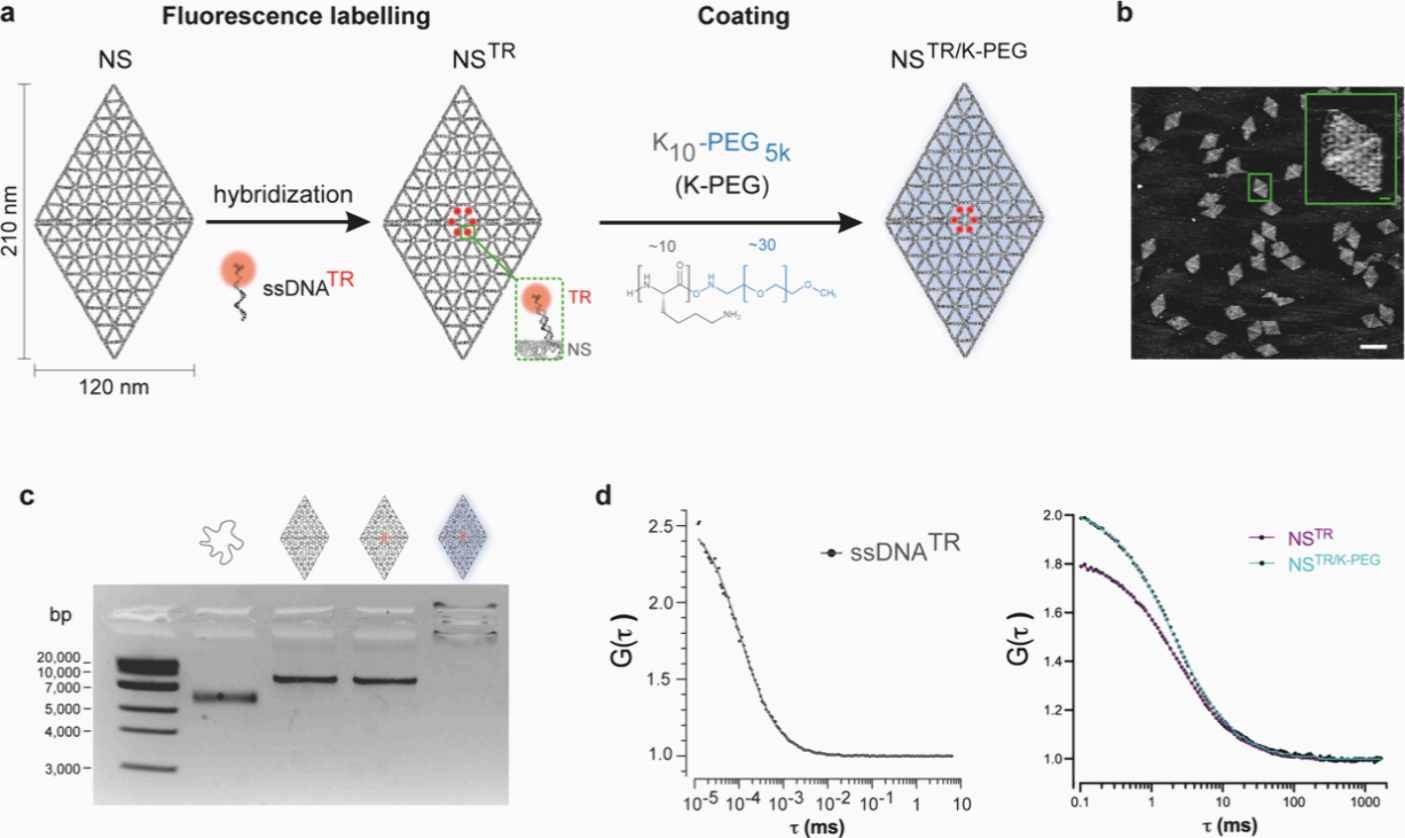
**Characterization of DNA origami NanoSheets.** (a) Schematic representation of fluorescence labeling and coating strategy of NanoSheets using Texas Red (TR) and K_10_-PEG_5K_ (K-PEG), respectively. NS: NanoSheet, NS^TR^: NanoSheet labeled with Texas Red, NS^TR/K‑PEG^: NanoSheet labeled with Texas Red and coated with K_10_-PEG_5K_, ssDNA^TR^: single strand DNA oligo conjugated with Texas Red. (b) AFM phase imaging of NS nanostructures. Magnified NS image shown in the inset. Scale bar: 200 nm, scale bar inset: 20 nm. (c) Analysis by agarose gel electrophoresis of the scaffold strand, NS, NS^TR^, and NS^TR/K‑PEG^ nanostructures. (d) Characterization of ssDNA^TR^, NS^TR^ and NS^TR/K‑PEG^ in solution by FCS. Autocorrelation curves, where the dots represent raw data and the solid lines correspond to one-component fit modeling. ssDNA^TR^: *n* = 6 technical repeats, total 20 s traces; NS^TR^: *n* = 24 technical repeats each, total 5 s traces and NS^TR/K‑PEG^: *n* = 24 technical repeats, total 5 s traces. The estimated diffusion time (τ_D_) for ssDNA^TR^ was 115 μs corresponding to a diffusion coefficient, D_ssDNA_^TR^, of 135 μm^2^/s. The estimated τ_D_ were 2.34 ± 0.36 ms for NS^TR^ and 2.02 ± 0.46 ms for NS^TR/K‑PEG^ (mean ± SD), corresponding to D_NS_^TR^ = 6.68 μm^2^/s and D_NS_^TR/K‑PEG^ = 7.73 μm^2^/s, respectively.

We performed fluorescence correlation spectroscopy (FCS), a single molecule detection method, to characterize NS properties and aggregation state in solution. For both NS^TR^ and NS^TR/K‑PEG^ samples, the curve of a one-component model fitted the measured data, indicating minimal aggregation (Figure 1d). The FCS analyses revealed a slightly longer diffusion time for NS^TR^ compared to NS^TR/K‑PEG^ (τ_D_ = 2.34 ± 0.36 ms and τ_D_ = 2.02 ± 0.46 ms, respectively), suggesting that the coated structures had a higher diffusion coefficient compared to the noncoated (Figure 1d). This is in accordance with the TEM results and could be an effect of the K-PEG shielding the electrostatic forces from the negatively charged DNA, resulting in a change of the shape of the structure. FCS analyses of ssDNA strands conjugated with Texas Red (ssDNA^TR^) yielded significantly shorter diffusion times compared to NS^TR^ and NS^TR/K‑PEG^ (Figure 1d), consistent with the lower molecular weight of ssDNA compared to NS. The average intensities of NS^TR^ and NS^TR/K‑PEG^ were 7.1 kHz/molecule, resulting in an estimated approximately two fluorophores per NS, as the average intensity of ssDNA^TR^ was 3.6 kHz/molecule. This estimate is lower than that obtained by the microplate fluorescence assay but, importantly, both methods provided comparable number of fluorophores for NS^TR^ and NS^TR/K‑PEG^.

### Temporal Profiles of NanoSheet Distribution in Zebrafish Embryos

To investigate the biodistribution profiles of NS^TR^ and NS^TR/K‑PEG^ *in vivo*, the wireframe DNA nanosheets were intravenously injected into the bloodstream of zebrafish embryos at 2 days post fertilization (dpf) (Figure 2a). We used the zebrafish model *Tg­(fli1:EGFP),* where the expression of green fluorescent protein (EGFP) is under the control of the *fli1* promoter (Figure 2b and Figure S4a). This zebrafish model enables visualization of the vasculature of the embryo and additionally labels hematopoietic cell populations as well as cranial neural crest cells in the pharyngeal arches. Live embryos were imaged with light sheet fluorescence microscopy (LSFM) for a period of 4 h, starting at 0.25 h post injection (hpi) (Figure 2c and Figure S4b). We observed that the fluorescence signal in the NS^TR^-injected embryos rapidly accumulated in the caudal hematopoietic tissue (CHT) (Figure S4b), which is functionally equivalent to the fetal liver of mammals, responsible for embryonic hematopoiesis and the resident site of scavenger endothelial cells.^41^ At the whole animal level, the signal in embryos injected with NS^TR/K‑PEG^ showed a different biodistribution profile compared to those injected with NS^TR^, with signal detected outside the vasculature (Figure S4b). At 4 hpi, NS^TR/K‑PEG^ signal was mainly localized to the CHT but was also detected in other regions of the embryo, such as brain and muscle (Figure S4b). Using LSFM, the average fluorescence intensity of circulating fluorescence signal in the lumen of the dorsal aorta (DA) showed no differences in embryos injected with NS^TR^ or NS^TR/K‑PEG^, due to low signal detection (Figure 2d). However, the detection limit of the fluorescence signal in live imaging using LSFM is affected by the comparatively low concentration of the fluorophore in circulation as well as by motion blur due to the blood flow, potentially accounting for the low signal detected in circulation.

**Figure 2:**
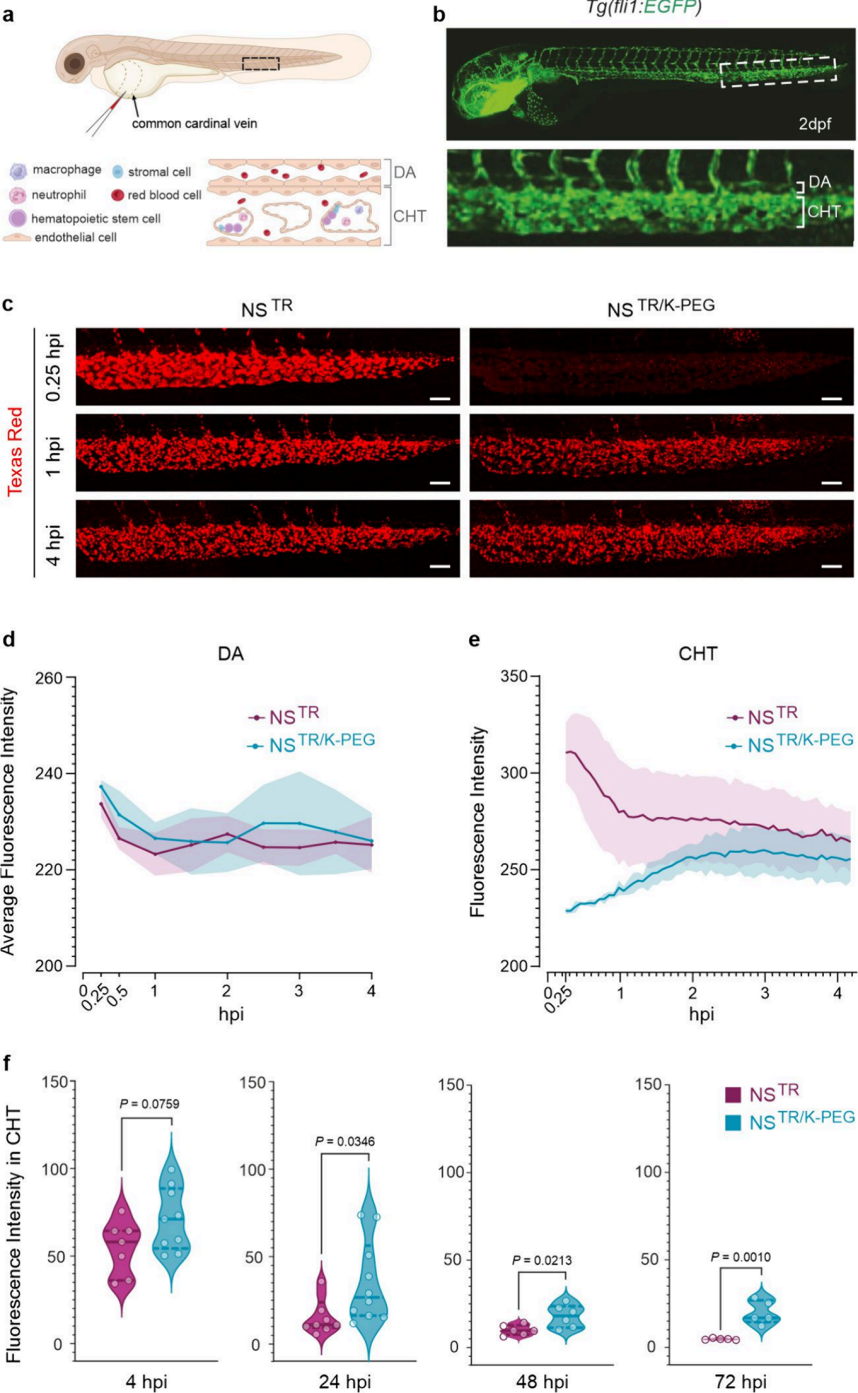
**Biodistribution profiles of intravenously injected NanoSheets in zebrafish embryos.** (a) Schematic showing the site of microinjection (common cardinal vain) in a 2 day post fertilization (dpf) zebrafish embryo. Boxed region illustrating the cell types forming the dorsal aorta (DA) and caudal hematopoietic tissue (CHT) of the embryo. (b) Whole-embryo view of a transgenic *Tg­(fli1:EGFP)* zebrafish embryo, showing the vasculature (green) at 2 dpf. Magnified region corresponding to the dashed box, showing the CHT below the DA. (c) Light sheet fluorescence microscopy (LSFM) imaging of Texas Red signal in live embryos, showing the accumulation of the NS^TR^ and NS^TR/K‑PEG^ in the CHT over time. hpi: hours post injection. Scale bar: 50 μm. (d) Quantification of Texas Red mean fluorescence in the DA lumen from LSFM imaging of embryos injected NS^TR^ or NS^TR/K‑PEG^ nanostructures at 0.25, 0.5, 1, 1.5, 2, 2.5, 3, 3.5, and 4 hpi. Values presented as average intensity from three different regions along the DA for each embryo at indicated time point (see Methods). *n* = 3 embryos per condition. Injection of each embryo was performed as an independent experiment. Values presented as mean ± SD (e) Quantification of Texas Red fluorescence intensity signal from 0.25 to 4 hpi with LSFM imaging at the CHT in embryos injected with NS^TR^ or NS^TR/K‑PEG^ nanostructures. *n* = 3 embryos per condition. Injection of each embryo was performed as an independent experiment. Values presented as mean ± SD (f) Quantification of Texas Red signal in the CHT of live embryos acquired by confocal imaging. Embryos were injected with NS^TR^ or NS^TR/K‑PEG^ structures and analyzed at 4, 24, 48, and 72 hpi. *n* = 5–10 imaged embryos per group, different embryos were imaged at each time point. *P*-values determined by parametric unpaired *t* test.

To investigate the clearance dynamics of the fluorescence signal in the CHT, we determined the mean fluorescence intensity over time in embryos injected with NS^TR^ or NS^TR/K‑PEG^ (Figure 2e). The signal intensity in the zebrafish embryos injected with NS^TR^ decreased substantially up to 1 hpi and continued to decrease gradually until 4 hpi (Figure 2c,e). In contrast, the zebrafish embryos injected with NS^TR/K-PEG^ showed moderate accumulation of fluorescence signal in the CHT, with the highest intensity detected at 2 hpi (Figure 2c,e). We further investigated the fluorescence intensity profiles in embryos injected with NS^TR^ and NS^TR/K‑PEG^ at the CHT by confocal imaging for up to 72 hpi. We observed that the fluorescence intensity decreased over time for both coated and noncoated structures, but was significantly higher in embryos injected with NS^TR/K-PEG^ compared to NS^TR^ at 24, 48, and 72 hpi (Figure 2f, Figure S5). Therefore, our results suggest that the K-PEG coating resulted in pronounced stabilization of the fluorescence signal in the CHT (Figure 2f). To investigate potential accumulation of fluorescence signal localization to the pronephric tubes of zebrafish embryos, we used the *Tg­(foxj1a:EGFP)* transgenic line, as previous studies have shown the importance of kidney clearance of DNA origami in mice.^18,19^ We observed that there was no accumulation of fluorescence signal in the pronephric tubes at 4 and 24 hpi, using confocal microscopy (Figure S6). Together, these results suggest that the K-PEG coating does not prevent fluorescence signal from accumulating in the CHT, the embryonic liver of zebrafish. However, there was a time window of 1 h before we detected accumulation of the fluorescence signal in embryos injected with NS^TR/K-PEG^ in the CHT whereas in embryos injected with NS^TR^ fluorescent signal was detected shortly after injection took place. Additionally, K-PEG coating was associated with a prolongation of the fluorescence signal at the CHT.

### Dynamics of NanoSheet Clearance at the CHT

After identifying CHT as the primary tissue for NS^TR^ and NS^TR/K‑PEG^ signal accumulation, we used Airyscan imaging to dissect its role in NanoSheet clearance in *Tg­(fli1:EGFP)* embryos, with subcellular resolution. Airyscan imaging was selected as it enables fast, super-resolution confocal imaging of dynamic processes with subcellular resolution *in vivo*. We focused on the time window from 0.5 hpi to 1.5 hpi, when the fluorescence intensity curves of embryos injected with NS^TR^ and NS^TR/K-PEG^ were substantially different (Figure 2e). Additionally, we imaged the dorsal aorta (DA) of live injected embryos (Figure 3a). Quantification of the Texas Red signal in EGFP^+^ cells in the CHT revealed that the signal intensity in NS^TR^–injected embryos accumulated and degraded faster than in NS^TR/K‑PEG^–injected embryos (Figure 3b). In addition, Texas Red signal was also detected in EGFP^–^ regions in embryos injected with NS^TR/K‑PEG^, indicating interaction with nonendothelial CHT resident cells (Figure S7). Signal from NS^TR/K‑PEG^ was detected in the circulation for the duration of the imaging, while signal from NS^TR^ was not detected by 1.5 hpi (Figure S7). To further investigate the aggregation state of NS^TR/K‑PEG^ in circulation, we performed FCS in the bloodstream of live zebrafish embryos. We measured the fluorescence intensity fluctuations of NS^TR/K‑PEG^ diffusing through a detection volume in the lumen of the DA (Figure 3c). Brightness analyses of the objects showed that coated NanoSheets started to form aggregates in circulation at 2 hpi, which increased in size with time (Figure 3d). Together, these data suggest that the endothelial cells within the CHT are the primary cell-type that interacts with fluorescent signal in embryos injected with the NanoSheets. However, we observed that the K-PEG coating affected the clearance dynamics and that signal in embryos injected with NS^TR/K‑PEG^ colocalized with cells within the CHT other than the endothelial cells, unlike signal from NS^TR^. Further, in embryos injected with NS^TR/K‑PEG^, but not NS^TR^, fluorescence signal was detected in circulation 1.5 h after the microinjections.

**Figure 3:**
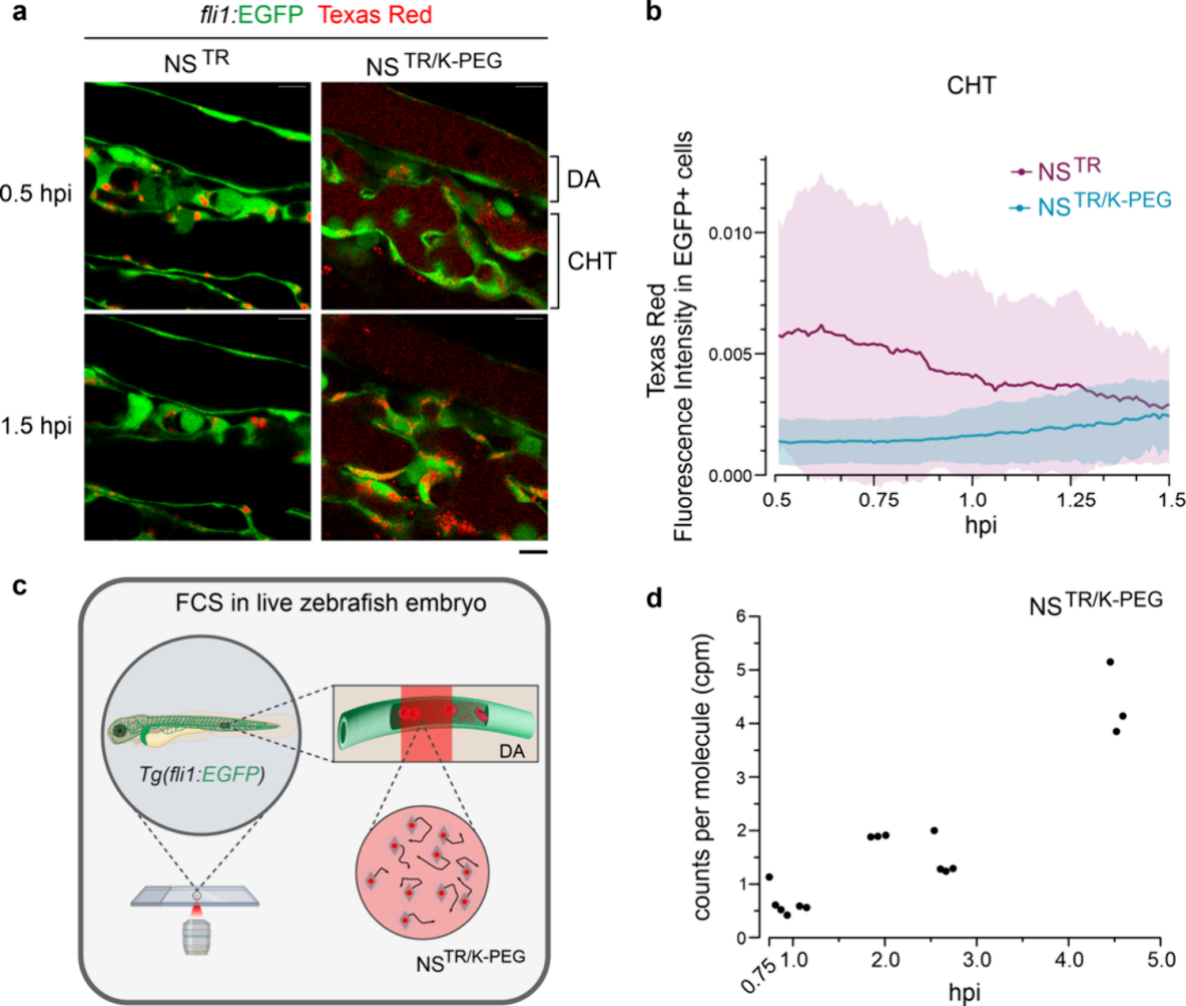
**NanoSheet-cell interactions at the CHT.** (a) Lateral view images of the DA and CHT in live *Tg­(fli1:EGFP)* embryos injected with NS^TR^ or NS^TR/K‑PEG^. Interactions of NanoSheets with endothelial cells were recorded from 0.5 to 1.5 hpi in 30 s intervals. *n* = 5 injected embryos per condition. Scale bar 10 μm. (b) Quantification of the NS^TR^ and NS^TR/K‑PEG^ levels in EGFP^+^ cells based on Texas Red mean fluorescence intensity values from 0.5 hpi to 1.5 hpi. Values presented as mean ± SEM (c) Schematic of the *in vivo* FCS experiment: a live *Tg­(fli1:EGFP)* embryo was injected with NS^TR/K‑PEG^ and immobilized on a glass surface with low melting point agarose, followed by FCS measurements of the Texas Red signal originating from the lumen of the DA. (d) Analysis of the brightness levels of Texas Red signal in live zebrafish embryos injected with NS^TR/K‑PEG^, at the DA at the indicated time points. Brightness levels (cpm: counts per molecule) was measured by FCS starting at 0.75 hpi. *n* = 9 technical repeats, total 20 s traces. The increase in cpm with time observed is likely underestimated due to fluorophore quenching in the aggregates.

### Whole Embryo Transcriptome Analysis of Texas Red-Labeled Cells

To identify the effects of the K-PEG coating on the cell types that became Texas Red-labeled following embryo injection with NS^TR^ or NS^TR/K‑PEG^ at the whole organism level with single-cell resolution, we performed single-cell RNA sequencing (scRNAseq). Embryos were injected at 2 dpf and dissociated into single cell suspensions at 4 hpi (Figure 4a), with approximately 200 injected embryos per sample. Texas Red^+^ cells were sorted by flow cytometry, for both NS^TR^ and NS^TR/K‑PEG^ injected embryos (Figure S8). After sequencing, we obtained 1048 Texas Red^+^ cells from NS^TR^ injected embryos with a median of 1908 genes per cell, and 1837 Texas Red^+^ cells from NS^TR/K‑PEG^ injected embryos with a median of 2076 genes per cell. We used UMAP dimensionality reduction for visualization of the clusters (Figure 4b). Based on previously annotated data,^42,43^ we identified ten different cell types for both conditions (Figure 4c). Further, we confirmed the identity of the cell types by comparing the analyzed genes to known cell markers (Figure 4d). Interestingly, 48.14% of the total number of Texas Red^+^ cells from embryos injected with NS^TR^ was identified as scavenger/vascular endothelial cells, whereas this percentage was only 14.92% in the embryos injected with NS^TR/K‑PEG^ (Figure 4e, Table S1). The largest group of Texas Red^+^ cells from NS^TR/K‑PEG^- injected embryos were identified as mesenchymal neural crest cells, constituting 31.74% of the total population (Figure 4e, Table S1). In addition, the percentages of cells identified as embryonic brain, red blood cells (RBC), the musculature system, and muscle cells were higher in embryos injected with NS^TR/K‑PEG^ compared to NS^TR^ (Figure 4e, Table S1). The percentage of immune cells was low in both conditions at this time point (4hpi) but was more than 2-fold higher for labeled cells from NS^TR^- than NS^TR/K‑PEG^- injected embryos (Figure 4e, Table S1). To assess potential transcriptional changes induced by K-PEG, we analyzed differential gene expression in scavenger/vascular endothelial cells between embryos injected with NS^TR^ and NS^TR/K‑PEG^. We identified only seven differentially expressed genes (Figure S9) with no significant pathway enrichment, suggesting that K-PEG does not elicit a notable transcriptional response. Together, these data indicate K-PEG coating allows for a broader distribution of the fluorescence signal at a whole organism level, with labeled cells present at higher percentages in tissues other than the CHT.

**Figure 4:**
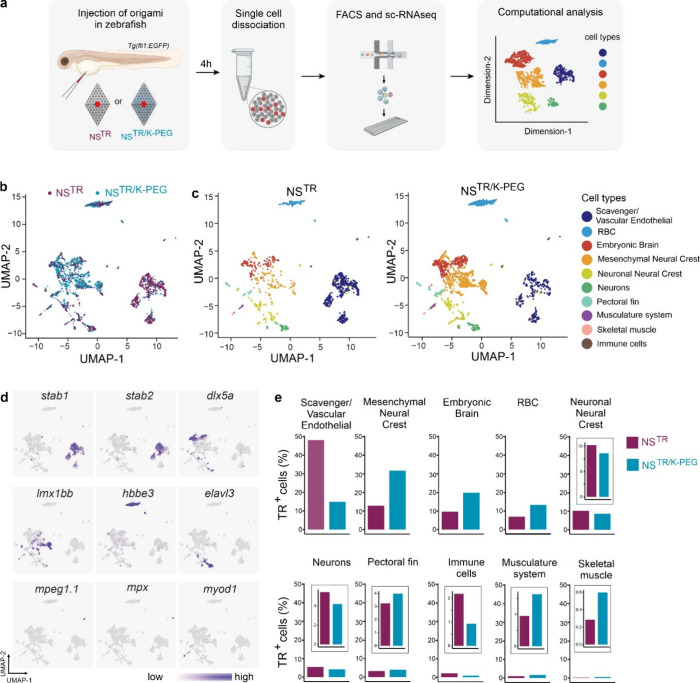
**Identification of Texas Red** ^**+**^ **cell types in zebrafish embryos after NanoSheet injection by single-cell RNA-seq** (a) Schematic overview of the strategy to identify cells labeled with Texas Red^+^ signal in zebrafish embryos injected with NanoSheets. *Tg­(fli1:EGFP)* embryos were injected with NS^TR^ or NS^TR/K‑PEG^ at 2 dpf. At 4 hpi, embryos were dissociated to single cells, Texas Red^+^ (TR^+^) cells were collected by flow cytometry and processed on a 10X platform for single-cell RNA profiling. (b) UMAP plots of 1048 TR^+^ cells from NS^TR^ and 1837 TR^+^ cells from NS^TR/K‑PEG^ injected embryos. (c) UMAP plots of identified cell types in NS^TR^ and NS^TR/K‑PEG^ samples. Ten different cell type clusters were identified for both NS^TR^ and NS^TR/K‑PEG^. (d) UMAP plots showing the relative expression levels of selected marker genes for scavenger endothelial cells (*stab1* and *stab2*), mesenchymal neural crest (*dlx5a*), embryonic brain (*lmx1bb*), red blood cells (RBC) (*hbbe3*), neuronal neural crest (*elavl3*), immune cells (*mpeg, mpx*) and skeletal muscle (*myod1*). (e) Percentages of identified cell-types labeled with NS^TR^ or NS^TR/K‑PEG^.

### Macrophage Contribution to NanoSheet Clearance

It has been previously reported that liver macrophages play a significant role in the clearance dynamics of nanoparticles in zebrafish.^44^ However, our findings from the whole-body single-cell transcriptome analysis of zebrafish embryos at 4 hpi, indicated that few macrophages (cells expressing the macrophage specific gene marker *mpeg1*, Figure 4e) were Texas Red^+^ after injection of the NanoSheets. As the number of macrophages increases over time after fertilization, we aimed to investigate the functional roles of macrophages within CHT in the clearance dynamics of fluorescence intensity over time in embryos injected with NanoSheets. We used a transgenic zebrafish line engineered for conditionally targeted ablation of macrophages^45,46^ (Figure 5a). Additionally, this transgenic zebrafish line expresses mCherry in macrophages which was used for their visualization, and therefore uncoated and coated NanoSheets were labeled instead with Alexa Fluor 488 and named NS^AF488^ and NS^AF488/K‑PEG^, respectively (Figure 5a). We induced macrophage ablation by treatment with metronidazole (MTZ), from 1 dpf and until the end of the experiment at 72 hpi. We observed a reduction in the numbers of macrophages detected on MTZ-treated embryos compared to nontreated controls, for the duration of the experiment (Figure 5b,c). We injected nonmacrophage-ablated or macrophage-ablated embryos with NS^AF488^ or NS^AF488/K‑PEG^ nanostructures at 2 dpf and measured their fluorescence intensity by confocal microscopy at 4, 24, 48, and 72 hpi. We confirmed that in this zebrafish model, the fluorescence intensity of signal in the CHT gradually decreased over time (Figure S10), consistent with the results we obtained for the *Tg­(fli1:EGFP)* line (Figure 2f). Interestingly, we observed that the fluorescence signal in embryos injected with NS^AF488^ was significantly higher in macrophage-ablated compared to nonablated embryos, at 48 hpi (Figure S10) and 72 hpi (Figure 5d) but not at earlier time points (Figure S10). In contrast, macrophage ablation did not significantly affect the fluorescence signal from NS^AF488/K‑PEG^ at any time points (Figure 5d and Figure S10), indicating that the removal of macrophages did not have a significant impact on the fluorescence intensity in embryos injected with NS^AF488/K‑PEG^. Together, these results suggest that the effects of CHT macrophages on the clearance of signal from uncoated NanoSheets start days after the injection of the nanostructures in the blood circulation of the zebrafish. In addition, our data suggests that K-PEG coating offers protection against macrophage-mediated clearance of fluorescence signal within the CHT.

**Figure 5:**
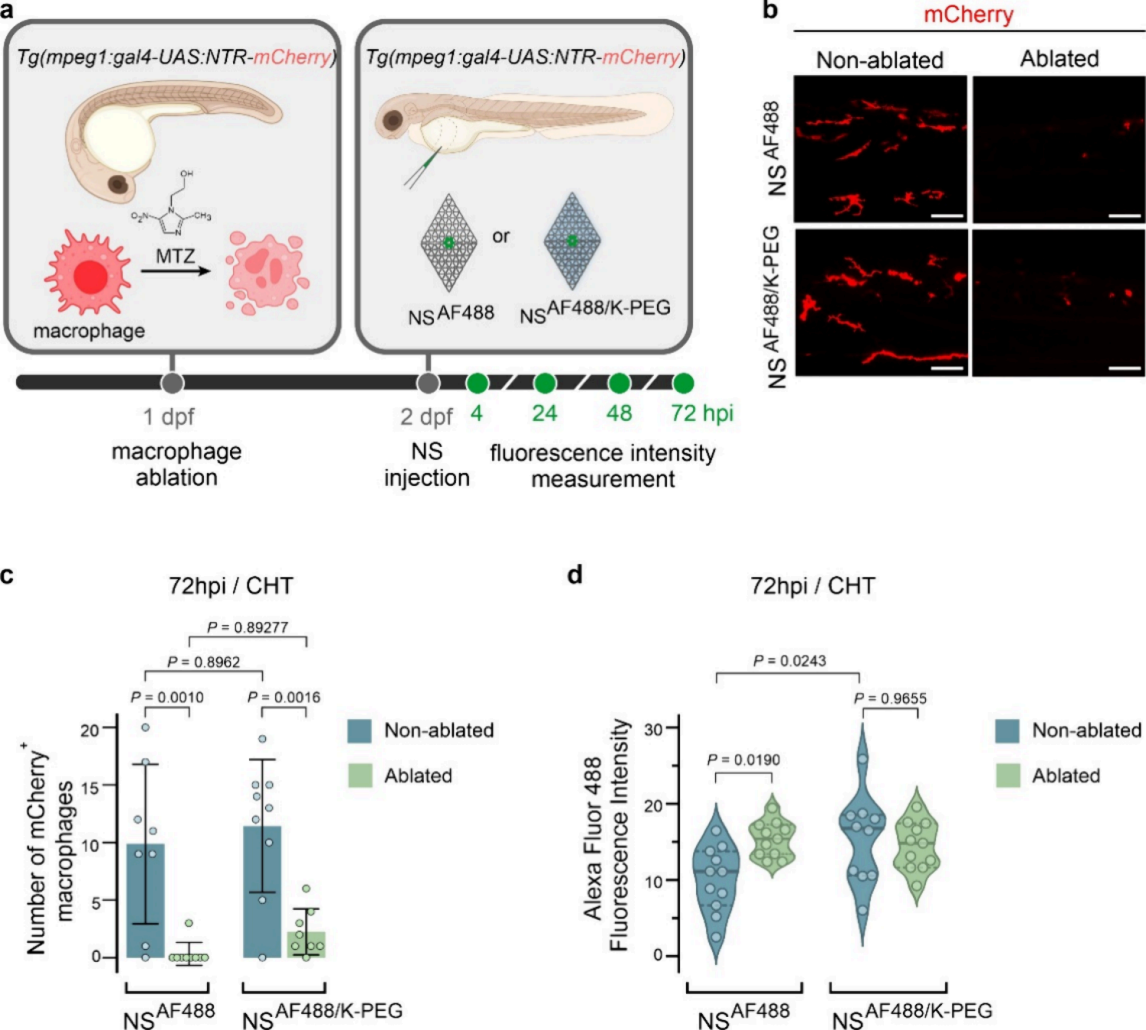
**Macrophage involvement on NanoSheet clearance.** (a) Schematic strategy to evaluate the role of macrophages on NanoSheet clearance in zebrafish embryos. *Tg­(mpeg1:gal4);Tg­(UAS:NTR-mCherry)* embryos were used, which express the nitroreductase enzyme (NTR) and the fluorescent marker mCherry under the control of the *mpeg1* promoter that drives gene expression in macrophages. mCherry labeling was used to detect macrophages. Targeted macrophage ablation is elicited by treatment with metronidazole (MTZ), which is converted by NTR into a cytotoxic byproduct that ablates macrophages. NanoSheets used in this assay were fluorescently labeled with Alexa Fluor 488 (AF488). Embryos were treated with MTZ from 1 day post fertilization (dpf) until the end of the experiment. Embryos were intravenously injected with NS^AF488^ or NS^AF488/K‑PEG^ 24 h later (2 dpf) and imaged by confocal microscopy at the CHT region at 4, 24, 48, and 72 hpi. Different embryos were imaged at each time point. (b) Live confocal microscopy imaging of the mCherry protein expressed in macrophages in the indicated conditions at 72 hpi. Scale bar 50 μm. (c) Bar plots of the quantifications of mCherry^+^ macrophages in CHT region at 72 hpi. Macrophage-ablated embryos were treated with MTZ from 1 dpf. Nonmacrophage-ablated embryos were not treated with MTZ. Values presented as mean ± SD *n* = 8 embryos (NS^AF488^ nonablated, NS^AF488/K‑PEG^ ablated) or *n* = 9 embryos (NS^AF488^-ablated, NS^AF488/K‑PEG^ nonablated) per condition. *P*-values determined by one-way ANOVA followed by Tukey’s multiple comparison test. (d) NS^AF488^ and NS^AF488/K‑PEG^ levels based on confocal AF488 mean fluorescence intensities of MTZ- treated and untreated larvae at 72 hpi. *n* = 10 embryos (NS^AF488/K‑PEG^ nonablated) and *n* = 11 embryos (NS^AF488^ ablated, NS^AF488^ nonablated, NS^AF488/K‑PEG^ ablated) per condition. *P*-values determined by one-way ANOVA followed by Tukey’s multiple comparison test.

## Conclusions

The DNA origami method allows for precise fabrication of nanostructures, offering control over their physical and chemical properties as well as site-specific functionalization with ligands of interest. These features position DNA origami nanostructures as promising therapeutic agents, capable of being selectively directed toward specific tissues. However, despite this potential, the evaluation of their performance within living organisms has been limited by the use of analyses methods that lack single cell resolution. To tackle this, we propose a strategy integrating DNA nanotechnology with embryonic zebrafish models, advanced microscopy, and single-cell RNA sequencing. This approach aims to provide a comprehensive understanding of the biodistribution and clearance dynamics of fluorescence intensity signal in embryos injected with fluorescently labeled wireframe DNA origami nanosheets in real-time under physiological conditions. Zebrafish embryos are less widely adopted as a model organism for studying the biodistribution and biological effects of nanomedicines *in vivo* compared to mice. This is often attributed to their lower physiological complexity, the absence of certain mammalian organs,^47^ and differences in functional and genetic similarity to humans.^48^ However, the rapid development and optical transparency of zebrafish embryos makes them particularly well-suited for high-throughput screening experiments.^49^

We imaged the fluorescence signal from live zebrafish embryo injected with fluorescently labeled wireframe DNA origami nanosheets and tracked signal patterns, distributions, and cell-interactions. Modifying the NanoSheet surface chemistry with K-PEG did not affect which tissues were labeled with fluorescence signal but had a large impact on its relative distribution in the different tissues. Thereby, this coating approach allowed for prolonged fluorescence signal interaction with tissues like the brain and musculature before their clearance. Furthermore, our observations highlighted the scavenger endothelial cells as the primary contributors to clearance of signal from NanoSheets injected samples at 2 dpf, with macrophages playing a role at later stages.

Here, we establish protocols for the use of the zebrafish embryo as a model system for assessing DNA origami distribution *in vivo*. While we demonstrated this approach using a single wireframe DNA origami structure, the methodologies presented here are broadly applicable for investigating a diverse range of DNA nanostructures, each with unique characteristics and advantages, including different types of wireframe DNA origami nanostructures. Furthermore, to investigate how the assembly of the DNA into DNA origami affects the biodistribution of fluorescence signal, future studies could include controls consisting of embryos injected with free DNA-fluorophore conjugates together with nonlabeled DNA nanostructures or with mixtures of scaffold and DNA staples not assembled into DNA nanostructures, with and without K-PEG coating. Finally, the K-PEG coating induced detectable conformational changes in the NanoSheets, as revealed by TEM imaging and FCS, underscoring the importance of further research on the effects of PEG coatings on the shape and *in vivo* functionality of DNA nanostructures. Thus, the use of zebrafish embryos with the protocols established in this paper could facilitate further research into the stability, targeting, and functional properties of various DNA origami designs and coatings, as well as enable high-resolution structural integrity analysis *in vivo* using FRET.

## Methods

### NS, NS^TR^, and NS^TR/K-PEG^ Production

The design-specific staple strands, ssDNA^TR^, and ssDNA^AF488^ (Supplemental Data 1) were ordered from Integrated DNA Technologies (IDT). The staple strands were ordered at a concentration of 100 μM and mixed to a final concentration of 463 nM. The scaffold strand p8064 was produced from modified m13 phage, as described before.^2^ For the synthesis of the NanoSheets, 10 nM of the scaffold was mixed with 100 nM of the appropriate staple mix in 1x PBS. The folding reaction took place in a thermocycler (MJ Research PTC/225 Gradient Thermal Cycler) by following the steps: annealing by heating to 80 °C for 5 min followed by a cooling to 60 °C over 20 min, then a slower cooling to 24 °C over 14 h, followed by removal of excess staples with 100 kDa MWCO Amicon centrifugal filters (Merck). For the NS^TR^ and NS^TR/K‑PEG^ designs, ssDNA^TR^ or ssDNA^AF488^ was mixed with the nanostructures at a 1:6 ratio and annealed in a thermocycler by heating to 37 °C for 1 h, cooling to 22 °C at 0.1 °C per min, incubating at 22 °C for 14 h and cooling to 4 °C at 0.1 °C per min. Excess ssDNA^TR^ or ssDNA^AF488^ was removed using 100 kDa MWCO Amicon centrifugal filters (Merck). To produce the NS^TR/K‑PEG^, NS^TR^ were mixed with oligolysine-PEG (K_10_-PEG_5K_, Alamanda Polymers) at a 1:1 ratio between the amines of lysines in K_10_-PEG_5K_ and the phosphates in DNA and incubated at RT for 30 min.^27^ The removal of the K_10_-PEG_5K_ coating was performed by treating the samples with chondroitin sulfate (Sigma- Aldrich) in 400x excess to the number of amines for 1 h at 37 °C. Quantification of the number of Texas Red fluorophores per NanoSheet was performed on a multimode microplate reader (Varioskan LUX).

To assess the stability of NS^TR^ and NS^TR/K‑PEG^ structures over time the samples were incubated for 72 h at 28 °C with DNase I (100, 10, 1, 0.1, or 0 U/mL) in 1X PBS. After the incubation, the DNase I was inactivated using 10 mM EDTA and 10% β -mercaptoethanol. The removal of the K_10_-PEG_5K_ coating was performed by treating the samples with chondroitin sulfate (Sigma- Aldrich) in 400x excess to the number of amines for 1 h at 37 °C and structure integrity was assessed by agarose gel elecrophoresis.

### AFM Imaging

An epoxy adhesive was used to glue a disc of mica to the center of a microscope slide. Using Reprorubber, a 3 cm high plastic ring was attached encircling the mica in order to form a chamber for imaging in liquid. NS were imaged by first diluting to 1 nM in TE-Mg buffer (5 mM Tris base, 1 mM EDTA, 10 mM MgCl_2_, pH 8.0) and pipetting 10 μL of the diluted nanostructures onto freshly cleaved mica. After 30 s, 4 μL of 5 mM NiSO_4_ was added and left to incubate at room temperature for another 4.5 min. Unattached nanostructures were removed by washing the mica with 1.0 mL of 0.1 μm-filtered TE-Mg buffer, and the chamber was then filled with 1.5 mL of filtered TE-Mg buffer prior to imaging. The imaging was performed in TE-Mg buffer using a JPK Instruments NanoWizard 3 Ultra atomic force microscope set to alternating contact (AC) mode using a Bruker AC40 cantilever.

### Agarose Gel Electrophoresis

After folding, NanoSheets were analyzed by running samples on 2% agarose (Thermo Scientific TopVision Agarose) gels (0.5x TBE buffer, 10 mM MgCl_2_ and 0.5 mg/mL ethidium or 1x SYBR Safe DNA stain) in an ice bath, for 2–4h at 75 V. The gels were imaged in the ImageQuant LAS 4000 system (GE Healthcare) or ChemiDoc MP Imaging System (Bio-Rad Laboratories).

### TEM Imaging

The NS and NS^K‑PEG^ were visually inspected by transmission electron microscopy (TEM) to assess integrity and monodispersity. Glow-discharged carbon-coated Formvar grids (Electron Microscopy Sciences) were used to apply 4 μL of 20 nM NS or NS^K‑PEG^ samples for 20 s at room temperature. Then, the liquid was blotted off on a filter paper and the excess was briefly washed with MiliQ water for 4 s. The samples were positively stained for 20 s with 2% (w/v) aqueous solution of uranyl formate with 20 mM NaOH. The uranyl formate was blotted off on a filter paper and washed with MiliQ water for 10 s. Then, the grids were air-dried for 1 h before imaging on a Talos 120C G2 operated at 120 kV with a Ceta-D detector at different magnifications in nanoprobe mode. Micrographs were further processed using Fiji v2.3.0.

### oxDNA Simulation

NS were designed using the vHelix program, a plugin for Autodesk Maya. The designs were converted to the oxDNA format using the tacoxDNA Web site (http://tacoxdna.sissa.it/) and analyzed using oxDNA coarse-grained modeling through the browser-based platform at https://oxdna.org/. Simulations of the nanostructures were carried out on the oxdna.org web server with the following parameters: 37 °C, a salt parameter of 1, 1 × 10^8^ timesteps with a dt value of 0.0001, and a preliminary relaxation step with the default parameters. Visualizations and videos of the simulations were made using the oxView tool (https://sulcgroup.github.io/oxdna-viewer/).

### *In Vitro* FCS

ssDNA^TR^, NS^TR^ and NS^TR/K‑PEG^ samples were prepared at a final concentration of 10 nM in 1x PBS. A droplet of 2.5 μL was transferred on a glass coverslip (#1.5 glass bottom dishes, MatTek Corporation). FCS measurements were performed on a Zeiss 980 confocal laser scanning microscope equipped for FCS, with a Zeiss water immersion objective and C-Apochromat 40×/1.2 NA. All samples were excited at 561 nm and fluorescence emission was collected at 570–694 nm. The FCS detection volume was calibrated using Alexa 568 (the diffusion coefficient of Alexa 568 was estimated to D = 350 μm^2^/s by comparing with Alexa 488, D = 414 μm^2^/s,^50^ using 488 nm excitation in both cases, yielding ω=0.25 μm and V = 0.42 fL. Experiments were performed at 28 °C. Autocorrelation curves were generated from measurements of ssDNA^TR^: n= 6 technical repeats, total 20 s traces; NS^TR^, NS^TR/K-PEG^: n= 24 technical repeats each, total 5 s traces. One-component diffusion fit was used to estimate the diffusion times.

### Zebrafish Microinjections and Macrophage Ablation

Zebrafish were housed in self-cleaning 3.5 l tanks with a density of 5 fish per liter in a centralized recirculatory aquatic system (Tecniplast). Basic water parameters were continuously surveilled and automatically adjusted to a temperature of 28 °C; conductivity 1200 μS/cm, pH 7.5. Other chemical water parameters were checked minimum monthly. The lighting scheme was 14 h light/10 h dark with a 20 min dawn and dusk period. Any animals are imported to a physically separate quarantine unit from which only surface-disinfected eggs are transferred to the breeding colony barrier. Health monitoring was done through Charles River according to the FELASA-AALAS guidelines.^51^ *Mycobacterium chelonae* has been detected in sludge samples, ZfPV-1 in sentinel fish. Historically, Pseudoloma neurophilia had been detected in sentinels. Zebrafish embryos were staged as previously described.^52^ All husbandry procedures are defined in SOPs which are available together with the latest health monitoring reports on request. All experiments were performed on animals younger than 5 days and no ethical permit was required according to 2010/63/EU.

Previously established zebrafish transgenic lines were used in this study; *Tg­(fli1:EGFP)* ^*y1*^,^53^ *Tg­(foxj1a:EGFP)* ^54,55^ and *Tg­(mpeg1:GAL4);Tg­(UAS:NTR-mCherry).* ^45,46^

For injection into the bloodstream, 2 dpf embryos were manually dechorionated and anesthetized with MS-222 (Merck). Four nl of the sample were delivered into the common cardinal vein using microcapillaries (TW100–4, World Precision Instruments), which were pulled using a Sutter P1000 needle puller.

The samples for injections were prepared at a final concentration of 100 nM in 1x PBS for the NS^TR^ and NS^TR/K‑PEG^. The total blood volume for a 2 dpf zebrafish is 60–89 nl^56^ and the estimated concentration of the injected nanostructures in our assays is approximately 4–6 nM.

For macrophage ablation double transgenic embryos *Tg­(mpeg1:GAL4);Tg­(UAS:NTR-mCherry)* were exposed to 10 mM MTZ (Sigma-Aldrich) diluted in 1% DMSO (VWR) in anE3 medium supplemented with 30 mg/mL 1-phenyl-2-thiourea (PTU, Acros Organics) from 24 hpf. For the assigned ablated animal groups, medium was replaced daily with freshly prepared MTZ solution up until the last imaging time point at 72 h post injection (hpi). The quantification of macrophages was based on a scoring system with cells being categorized in three groups (type1: macrophages with long projections, type 2: macrophages with size μm in diameter, type 3 with size 10 μm in diameter and signs of morphological cell death). Type 1 and type 2 cells were included in the quantifications.

### Light Sheet Fluorescence Microscopy

*Tg­(fli1: EGFP)* embryos (48 hpf) were anesthetized in 0.005% tricaine (MS222) and mounted in 1% low melting point agarose (Thermo Scientific) in a glass capillary (50 μL, BRAND GmbH) directly after they were injected with NS^TR^ or NS^TR/K‑PEG^. The agarose cylinder was extruded into the sample chamber of a Light Sheet Z.1 microscope (Carl Zeiss, Germany) containing egg water solution (E3) at 28.5 °C. The 488 and 561 nm laser lines were used to excite fluorescence and images were acquired using a water dipping 10X detection objective (W-Plan-APOCHROMAT-0.5NA) and dual side 5X illumination objectives (LSFM-5*X*/0.1NA). Samples were illuminated from two sides and tiled (1 × 3), Z-stacks were acquired every 4 min for 4 h. The z-stacks were max intensity projected and stitched in Zen Blue 3.6 (Carl Zeiss, Germany). When required, a drift correction was applied to time-lapse images using the “Linear Stack Alignment with SIFT multichannel” plugin in Fiji. The maximum intensity projections of the *z-*stacks over time were analyzed with Fiji v2.14.0.^57,58^ The average intensity of NS^TR^ or NS^TR/K‑PEG^ was measured within a 10*10 μm rectangular area in the center of the lumen of dorsal aorta (DA) for the slice of each time point. This measurement was repeated in three different sites along the DA, in each embryo. The ROI selection of the CHT was performed based on anatomical analysis of EGFP+ signal to determine the boundaries of the CHT, so that the entirety of the CHT was imaged for each embryo. All the displayed images were acquired from the same experiments and their contrast values were adjusted for visualization purposes.

### *In Vivo* Confocal Imaging

Injected embryos were collected at different time points, anesthetized, and mounted in 1% methylcellulose (Sigma). The confocal images were acquired with a Leica TCS SP8 microscope and the LAS X software (v. 3.5.5.19976). The CHT of the animals was scanned with a × 40 water-immersion objective and the maximum intensity projections of the *z-*stacks were analyzed using Fiji v2.14.0.^57,58^ All the displayed images were acquired from the same experiments and their contrast values were adjusted for visualization purposes.

### *In Vivo* Airyscan Imaging

*Tg­(fli1:EGFP)* embryos (48hpf) were injected with NS^TR^ or NS^TR/K‑PEG^. Thirty minutes post injections one successfully injected embryo per group was anesthetized in 0.005% tricaine (MS222) and mounted on a lateral position in 1% low melting point agarose (Thermo Scientific) on a cover-glass bottomed Petri-dish (MatTek). Once the agarose had polymerized, the dish was filled with egg water solution (E3).

The zebrafish caudal hematopoietic tissue (CHT) was imaged on a Zeiss LSM980 Airyscan using a C-Apochromat 40*x*/1.2 NA water immersion objective equilibrated at 28.5 °C. Fluorescence was excited using the 488 and 561 laser lines and single-plane 2 color images were acquired every 30 s for 1 h in the Airyscan SR-4Y mode. Following the time-lapse acquisition, a Z-stack covering approximately 40 μm was acquired. Three dimensional reconstructions were made using Imaris v 9.7.0. Image analysis of the image intensities was performed using CellProfiler 4.2.6.^59^ For segmentation of the endothelial cell regions of the image, the green channel (EGFP is expressed under the control of the promoter for the endothelial marker Fli1a) was filtered using a median filter (filter radius 30 pixel) and then segmented using the Otsu threshold method within the Identify Primary Object module. The achieved objects were expanded by 6 pixels to ensure coverage of small intracellular vesicles. The mean intensities of the red channel (DNA origami) were then measured within the expanded EGFP^+^ regions. All the displayed images were acquired from the same experiments and their contrast values were adjusted for visualization purposes.

### *In Vivo* FCS Measurements Acquisition

*Tg­(fli1:EGFP)* zebrafish embryos (48 hpf) were injected with NS^TR/K‑PEG^. A successfully injected embryo was anesthetized and mounted as described above. FCS measurements were performed on a Zeiss 980 confocal laser scanning microscope equipped for FCS, with a Zeiss water immersion objective and C-Apochromat 40×/1.2 NA and equilibrated at 28.5 °C. Fluorescence of NS^TR/K-PEG^ was excited using the 561 nm laser line at the lumen of the dorsal aorta above the CHT. Measurements were recorded starting at 0,75 hpi and collected at 16 time-points during a 226 min period, and each measurement consisted of n = 9 technical repeats, total of 20s trace. The brightness in CPM (counts per molecule) for each measurement was calculated by dividing the average fluorescence intensity of the measurement with the number of particles (N) estimated through a fitted FCS curve. For all measurements the fluorescent spikes were clearly distinguishable, and no background correction was performed for any of the measurements as the background signal was too low to be quantified except in the final three measurements. If background correction was performed for the final three measurements, the CPM-values increased slightly.

### Statistical Analysis

Embryos were randomly assigned to the NS^TR^, NS^TR/K‑PEG^ injection and MTZ treatment groups. No statistical methods were used to predetermine sample size. Data collection and analysis were not performed blind to the conditions of the experiments. Graphs show all individual data points. Every embryo was injected with freshly prepared NanoSheets. For all experiments performed only successfully injected embryos were selected.

For light sheet and Airyscan imaging and for FCS analysis, several embryos were injected, one successfully injected embryo was selected and imaged in one experimental session at a time.

One-way ANOVA with Tukey’s multiple comparisons test was performed for the analysis of multiple groups, whereas parametric unpaired *t* test was used for analysis of pairwise significance. Statistical analysis and graphical representation of the data were performed with GraphPad Prism 10.0.3.

### Single-Cell RNA-seq Preparation

For single-cell RNA sequencing of TR^+^ cells, dissociation of embryos (48 hpf) into a single cell suspension was performed as previously described^60^ with minor modifications. Four hours post injections, around 200 *Tg­(fli1:EGFP)* embryos were euthanized in 0.02% tricaine and washed twice with 1X PBS. The tissue was dissociated with harsh pipetting in 0.25% trypsin-EDTA (GIBCO) and 100 mg/mL collagenase (Sigma-Aldrich) for 3 min at 30 °C, followed by resuspension in DMEM-10% FBS. The cells were then pelleted at 700xg for 5 min, washed once in 1X PBS, filtered through a 40 μm cell strainer (MERCK) and stained with 1 mM DAPI solution (Abcam). The flow cytometry sorting and analysis was performed at the Biomedicum Flow cytometry Core facility that receives funding from the Infrastructure Board at Karolinska Institutet. TR^+^ cells were FACS sorted on a FACSARIA III with BD FACSDIVA software v. 9.0.1 (BD Biosciences). Cells were initially identified by gating cells on FSC-A versus SSC-A, followed by gating on FSC-A versus FSC-H to detect singlets. Then live cells were identified by gating cells on DAPI versus SSC-A. We gated PE versus FITC-A to detect Texas Red+ cells in comparison to the GFP+ and GFP- populations, relative to the baseline signal for each sample. The GFP+ cells (endothelial cells) were identified by comparing FITC-A versus SSC-A. Note that Texas Red+ cells were included independently of GFP-signal. scRNaseq libraries were generated from the sorted cells using 10X Genomics Chromium Single Cell 3′ Reagent Kits v3.1 per manufacturer’s instructions, targeting 8000 cells. The cDNA libraries were amplified with 14 PCR cycles and sequenced on using NextSeq 500/550 High Output Kit v2.5 (150 Cycles) on NextSeq 550 platform (Illumina), with a read depth of at least 25 M reads per cell.

### Single-Cell RNA-seq Analysis

The sequencing data was processed and analyzed using the 10X Cell Ranger pipeline (v. 7.1.0)^61^ with the Danio rerio (GRCz11.105) genome and Seurat software package (v. 4.3.0)^62^ for R (v. 4.1.3). The average UMI count per gene was 1402 and the average dropout rate across the different cell types was 89%. Data was filtered to remove cells with low gene count (<200), high read count (>75’000), and high percentage mitochondrial genes (>20%). The data was normalized (NormalizeData) and scaled (ScaleData) for the 2000 most variable features (FindVariableFeatures). Subsequently, we performed dimensionality reduction of the data to 100 principal components, from which the top 50 was used to generate UMAP and tSNE visualizations. The samples were integrated to allow for better comparison (FindIntegrationAchors, IntegrateData), prior to clustering using k = 20 for the k-nearest neighbor algorithm and resolution 1.2 (FindNeighbors, FindClusters).

To annotate the clusters with their predicted cell type we analyzed the marker genes for each cluster (FindAllMarkers) using Wilcox Rank Sum test and compared these to known cell markers (Figure 4). In addition, we used an annotated cell atlas from Zebrahub^43^ (2 dpf, v. 1) (10.1101/2023.03.06.531398) together with the scPred package (v. 1.9.2)^63^ for training an algorithm using supervised learning, which was then used to annotate our data set. To annotate cranial neural crest-derived cells (CNCCs) we compared previously published and annotated markers^42^ to marker genes in clusters of progenitor cells. Taken together, the predictions from the different methods all contributed to the final cell type annotation. Differentially expressed genes in scavenger/vasculature endothelial cells from embryos injected with NS^TR/K‑PEG^ compared to NS^TR^ was identified (FindAllMarkers) using Wilcox Rank Sum test with a logFC of 0.2 and p-value of ≤ 0.05.

## Supplementary Material





## Data Availability

Code used for analysis of RNA-seq data is available at https://github.com/TeixeiraLab/Kolonelou-et-al

## Notes

Single-cell RNA-seq data files are available via the ArrayExpress database at https://www.ebi.ac.uk/biostudies/arrayexpress under accession number E-MTAB-13554. Reference Danio rerio (GRCz11.105) used for gene mapping in Cell Ranger (v. 7.1.0) (https://www.ensembl.org/Danio_rerio/Info/Index). Annotated single-cell RNA-seq atlas of zebrafish at 2 dpf (https://zebrahub.ds.czbiohub.org/data). All raw data files are available upon request.

## Notes

Nanosheet staples and ssDNA oligos (XLSX)
Simulation analysis of NS (Figure S1); Characterization of NS^TR^ and NS^TR/K‑PEG^ (Figure S2); Stability of NS^TR^ and NS^TR/K‑PEG^ in the presence of DNase I (Figure S3); Light sheet fluorescence microscopy images of live *Tg­(fli1:EGFP)* embryos injected with NS^TR^ and NS^TR/K‑PEG^ (Figure S4); Confocal imaging of the CHT over time (Figure S5); *In vivo* confocal imaging of *Tg­(foxj1a:EGFP)* embryos injected with NS^TR^ and NS^TR/K‑PEG^ (Figure S6); 3D rendering of z-stack Airyscan images of CHT and DA in *Tg­(fli1: EGFP)* embryos injected with NS^TR^ or NS^TR/K‑PEG^ at 1.5 hpi (Figure S7); Isolation of Texas Red-labeled cells (Figure S8); Differentially expressed genes of NS^TR/K‑PEG^ compared to NS^TR^ in scavenger/vasculature endothelial cells (Figure S9); Effect of macrophage ablation on the fluorescence intensity at the CHT of zebrafish injected with NS^AF488^ and NS^AF488/K‑PEG^ (Figure S10); Number of cells interacting with NS^TR^ or NS^TR/K‑PEG^ detected for each cell type from single-cell RNA-sequencing (Table S1) (PDF)
