# Supplementary material for: Effects of Oligolysine-Polyethylene Glycol Coating on the Biodistribution of Wireframe DNA Origami Nanosheets in Zebrafish Embryos
Source: ACS Nano. 2025 Sep 3;19(36):32145–57. doi: 10.1021/acsnano.5c05801 (PMC12444974; doi:10.1021/acsnano.5c05801)
Supplement: Supplementary file 2 [file nn5c05801_si_002.pdf]

Supporting Information

Effects of Oligolysine-Polyethylene Glycol Coating on the Biodistribution of Wireframe DNA Origami Nanosheets in Zebrafish Embryos

Christina Kolonelou[1], Enya Engström[1], Lars Bräutigam[2], Steven Edwards[3], José M. Dias[1,4], Joel Spratt[1], Christos Karampelias[4], Iris Rocamonde-Lago[5], Björn Högberg[5], Stefan Wennmalm[3], Hjalmar Brismar[3], Olov Andersson[4,6], Ana I. Teixeira[1,*]

[1]Department of Physiology and Pharmacology, Karolinska Institutet, Stockholm, 171 77, Sweden.

[2]Department of Comparative Medicine, Karolinska Institutet, Stockholm, 171 77, Sweden.

[3]Science for Life Laboratory, Department of Applied Physics, KTH Royal Institute of Technology, Solna, 171 21, Sweden.

[4]Department of Cell and Molecular Biology, Karolinska Institutet, Stockholm, 171 77, Sweden.

[5]Department of Medical Biochemistry and Biophysics, Karolinska Institutet, Stockholm, 171 77, Sweden.

[6]Department of Medical Cell Biology, Uppsala University, Uppsala, 751 23, Sweden.

*Corresponding author: ana.teixeira@ki.se

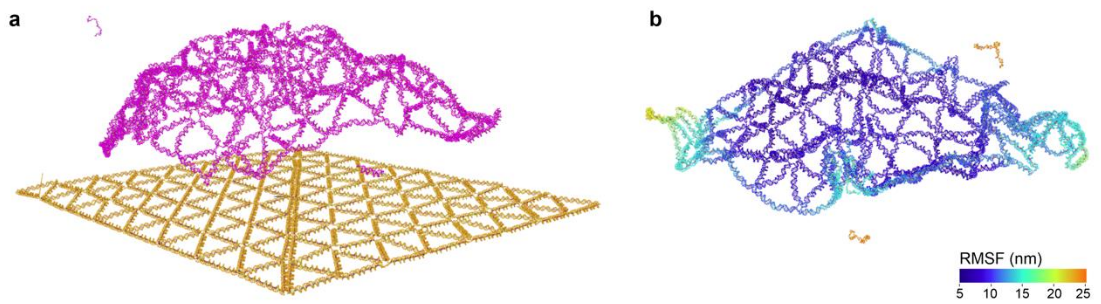

Figure S1. Simulation analysis of NS. (a) Comparison between the schematic illustration of the NS design (yellow) and a simulation snapshot of NS structure generated by the oxDNA software (https://oxdna.org/) (pink). (b) Rigidity analysis of NS with the oxDNA coarse-grained modelling software, showing a flexible structure. RMSF: root mean square fluctuation.

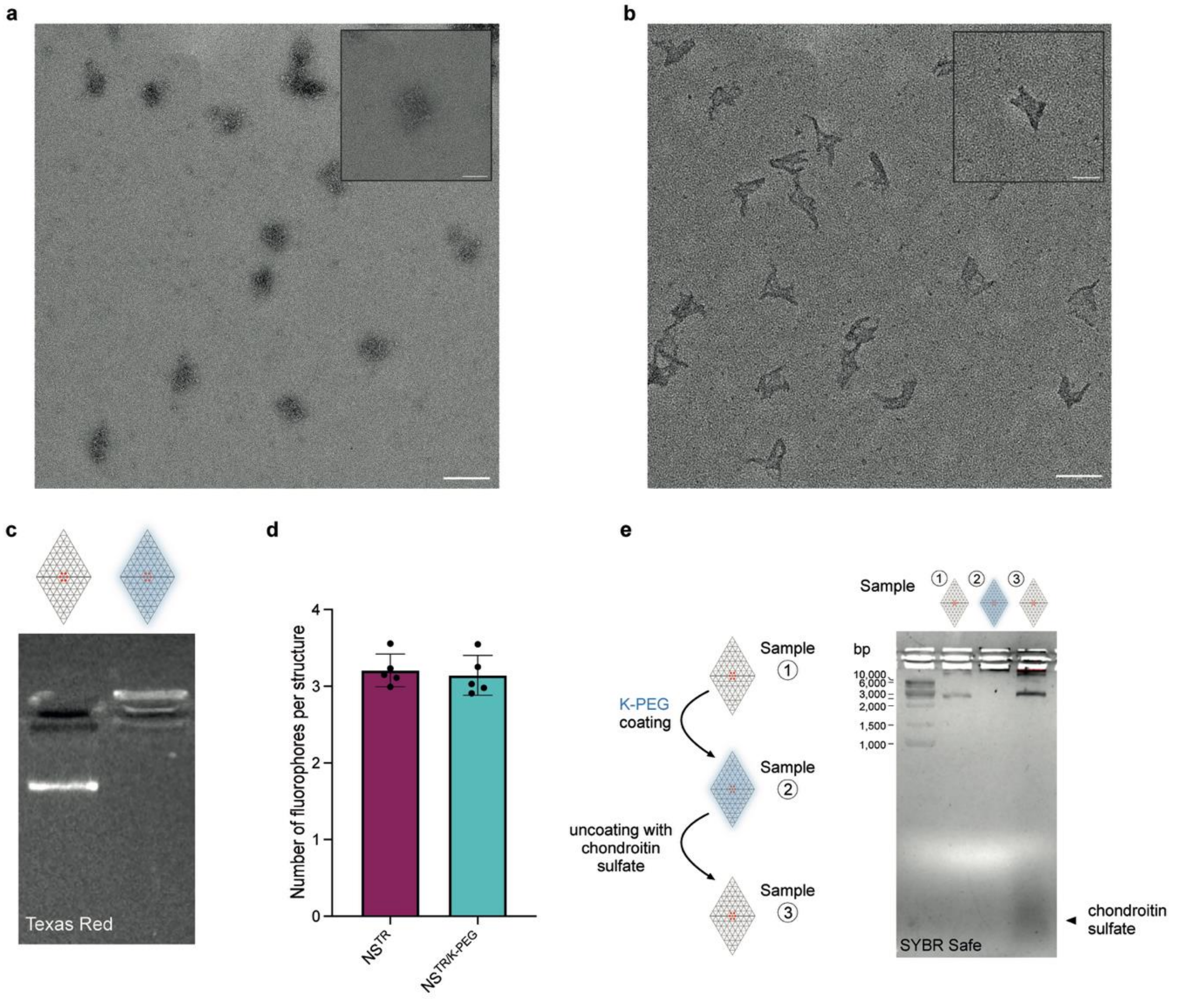

Figure S2. Characterization of NS^{TR} and NS$^{TR/K\text{-}PEG}$. Transmission electron microscopy (TEM) images of (a) NS and (b) NS$^{K\text{-}PEG}$ with positive staining. Insert: TEM images acquired with higher magnification. Scale bar: 100 nm, scale bar insert: 50 nm. (c) Analysis of NS^{TR} and NS$^{TR/K\text{-}PEG}$ by agarose gel electrophoresis. The gel was imaged using the dsCherry channel to detect the Texas Red signal. (d) Quantification of the number of TexasRed fluorophores per NanoSheet by measuring fluorescence using multimode microplate reader, total of 5 different conjugation replicates per condition. (e) Characterization of the coating strategy of NS by agarose gel electrophoresis. Coating of NS with K-PEG sequesters the negative charges associated with the NS, resulting in poor migration of the structures in agarose gel electrophoresis. Treatment with the negatively charged sulfated glycosaminoglycan chondroitin sulfate electrostatically sequesters the positively charged K-PEG and removes K-PEG from NS$^{TR/K\text{-}PEG}$, re-establishing the migration pattern of NS structure in agarose gel electrophoresis.

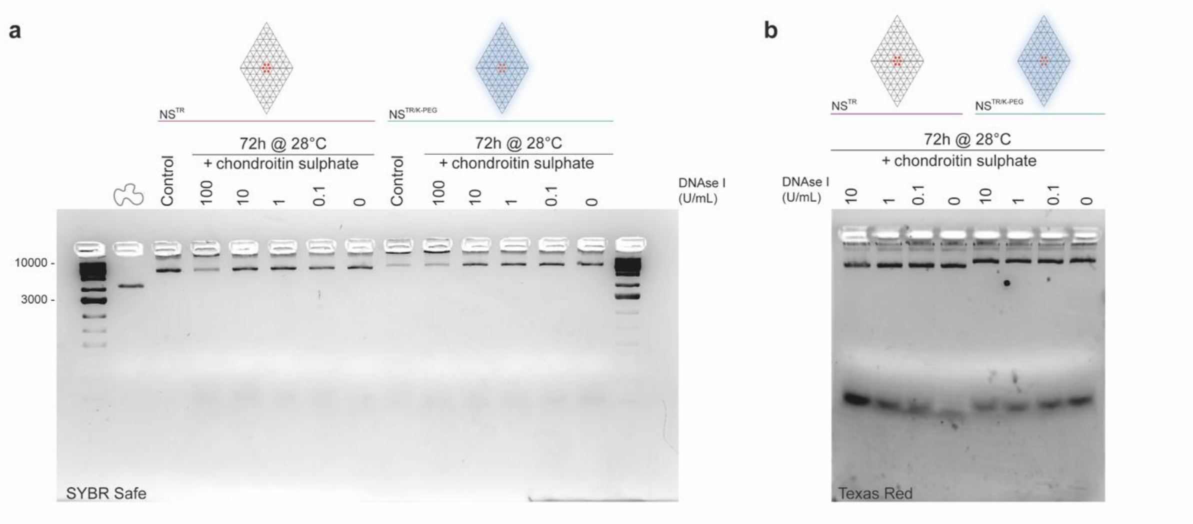

Figure S3. Stability of NS^{TR} and NS$^{TR/K\text{-}PEG}$ in the presence of DNAse I. Analysis by agarose gel electrophoresis of NS^{TR} and NS$^{TR/K\text{-}PEG}$ structures (10 nM) incubated with the indicated concentrations of DNAse I (U/mL) for 72 hours at 28°C. Gels imaged for (a) DNA and (b) dsCherry to detect Texas Red fluorophores. After incubation, the DNAse I was inactivated using 10 mM EDTA and 10 % β-mercaptoethanol and incubated with 400X excess of chondroitin sulphate prior to analysis, for both coated and uncoated structures. The scaffold was p8064 and control samples were freshly folded structures with and without K-PEG coating.

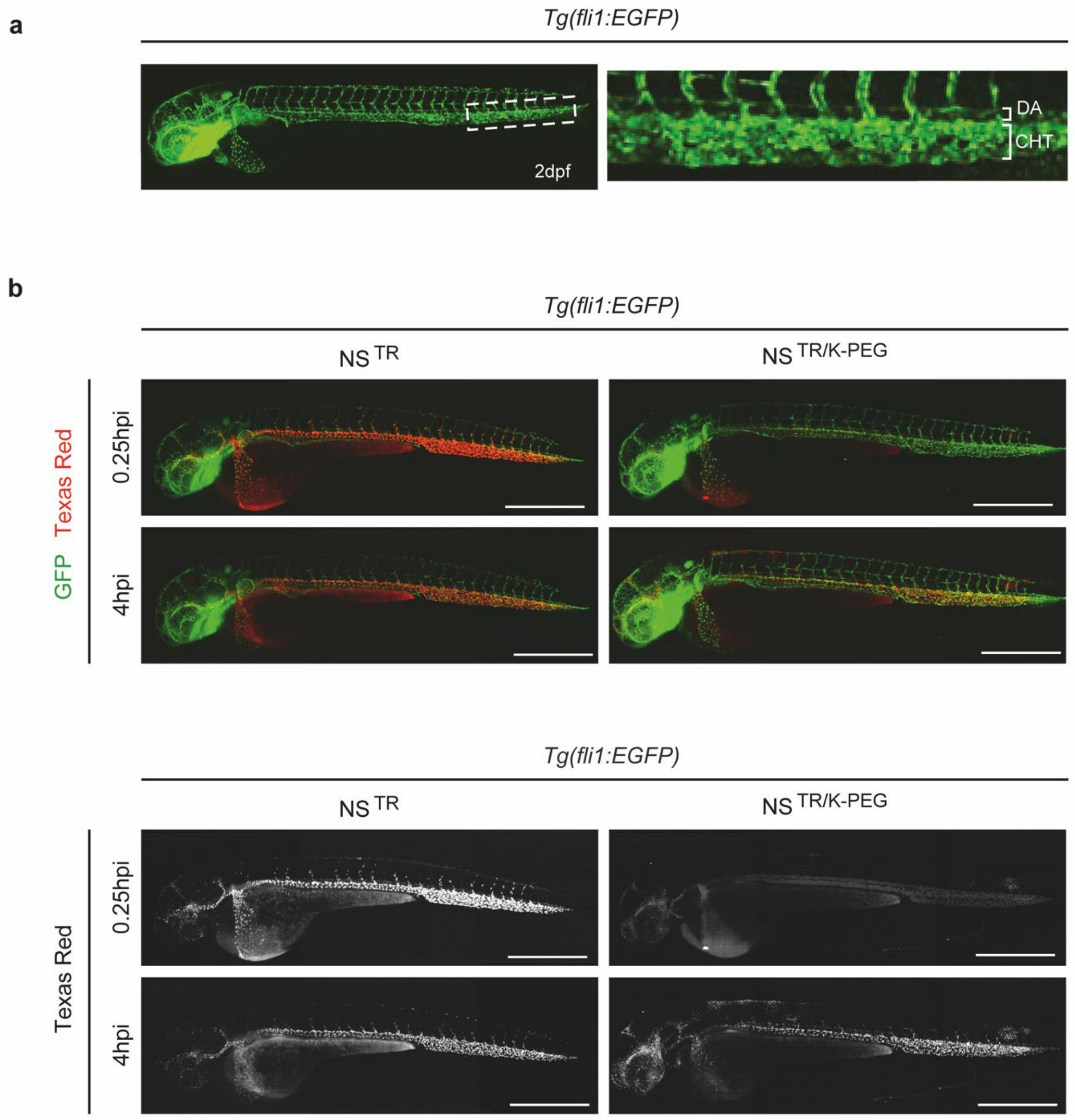

Figure S4. Light sheet fluorescence microscopy images of live *Tg(fli1:EGFP)* embryos injected with NS^{TR} and NS$^{TR/K\text{-}PEG}$. (a) Whole-embryo view of a transgenic Tg(fli1:EGFP) zebrafish embryo, showing the vasculature (green) at 2 dpf. Magnified region corresponding to the dashed box, showing the CHT below the DA. Repeated from Figure 2b. (b) Biodistribution profiles of NS^{TR} and NS$^{TR/K\text{-}PEG}$ at 0.25 and 4 hpi. Scale bar 500 µm.

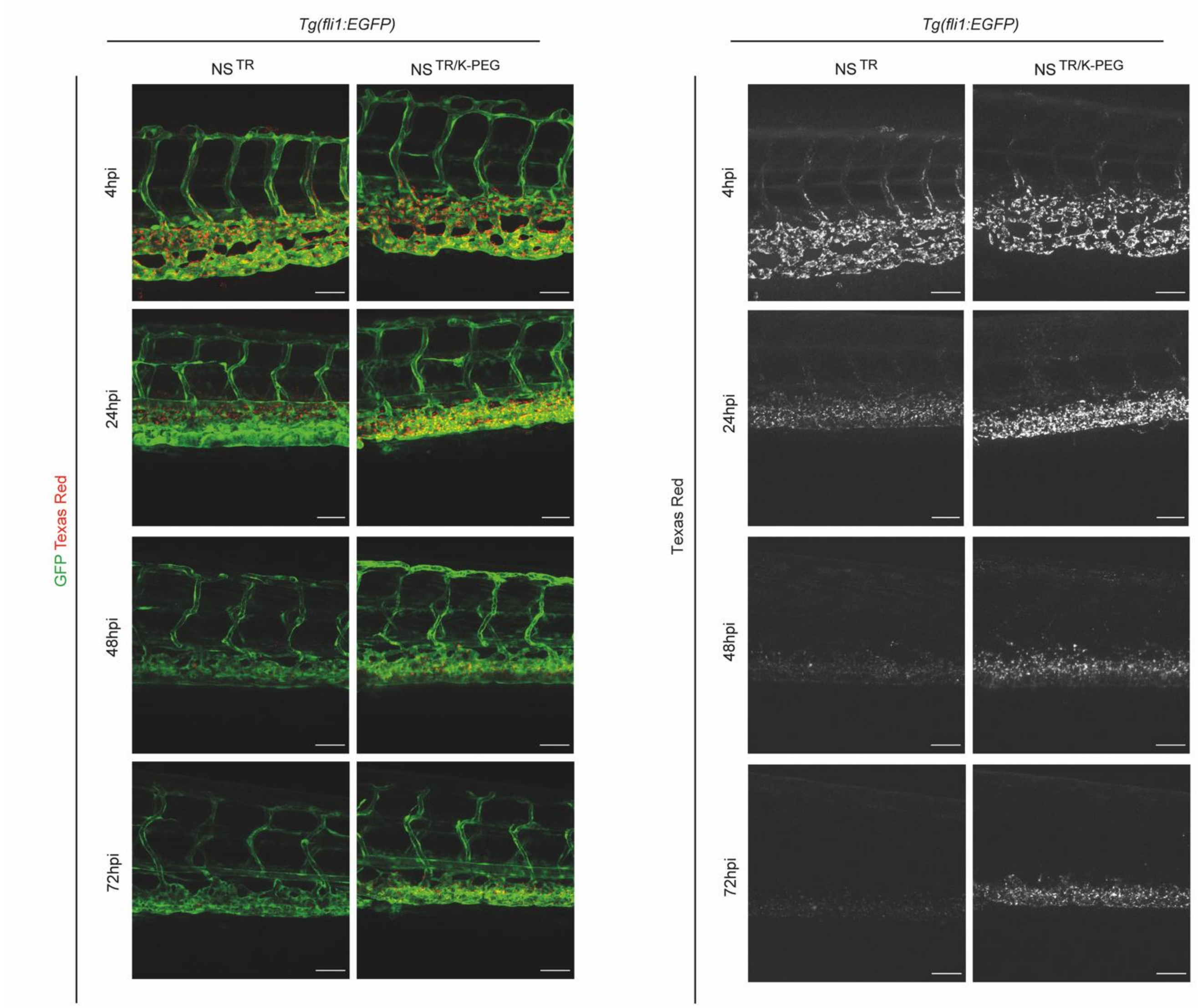

Figure S5. Confocal imaging of the CHT over time. Representative images from confocal imaging of the CHT at 4-, 24-, 48- and 72-hpi used to quantify Texas Red fluorescence intensity signal in the CHT of zebrafish embryos injected with NS^{TR} or $NS^{TR/K\text{-}PEG}$. Scale bar 50 μm.

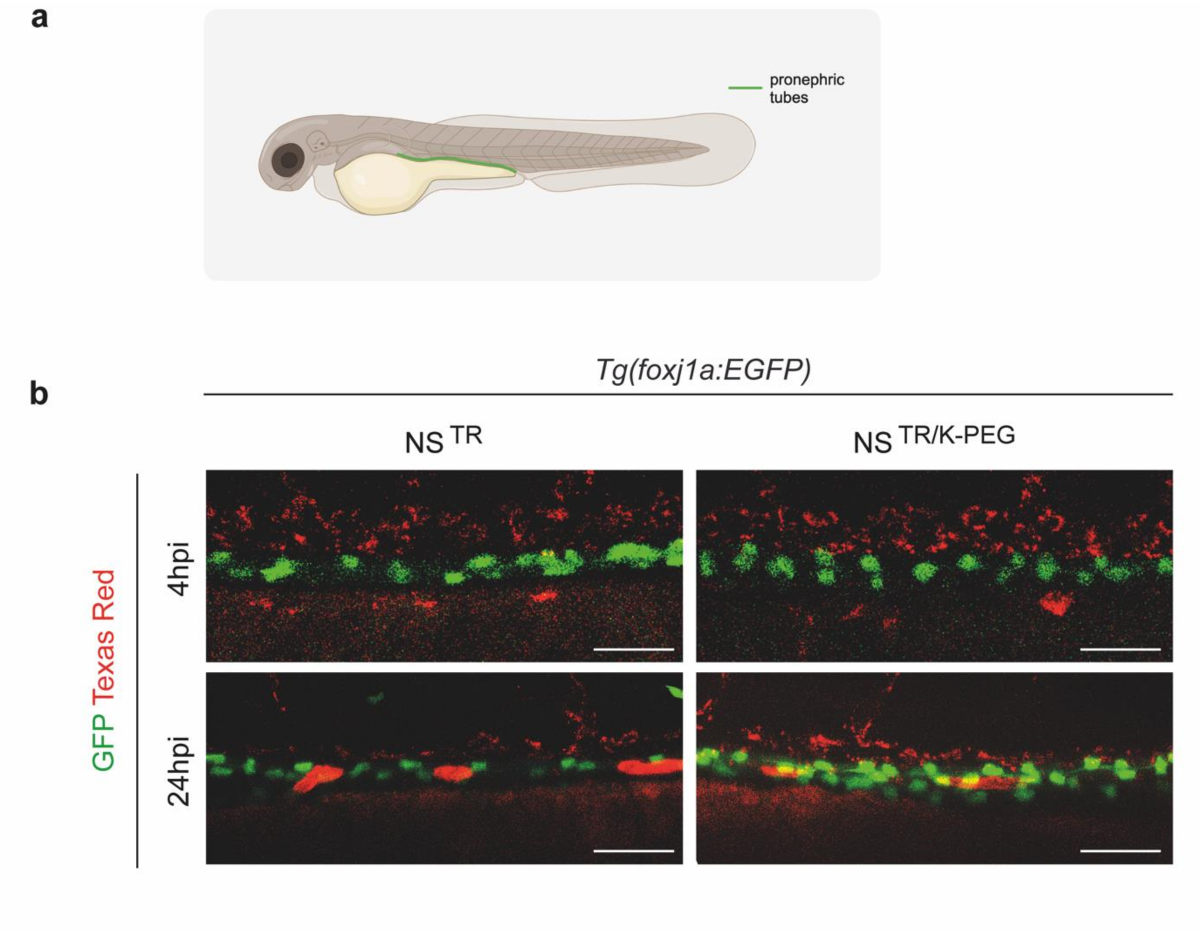

Figure S6. *In vivo* confocal imaging of *Tg(foxj1a:EGFP)* embryos injected with NS^{TR} and NS$^{TR/K\text{-}PEG}$. (a) Illustration of the location of pronephric tubes in zebrafish embryos. (b) Imaging of Texas red signal in selected regions of the pronephric tubes (green) in embryos injected with NS^{TR} and NS$^{TR/K\text{-}PEG}$ at 4 and 24 hpi. Scale bar 50 µm.

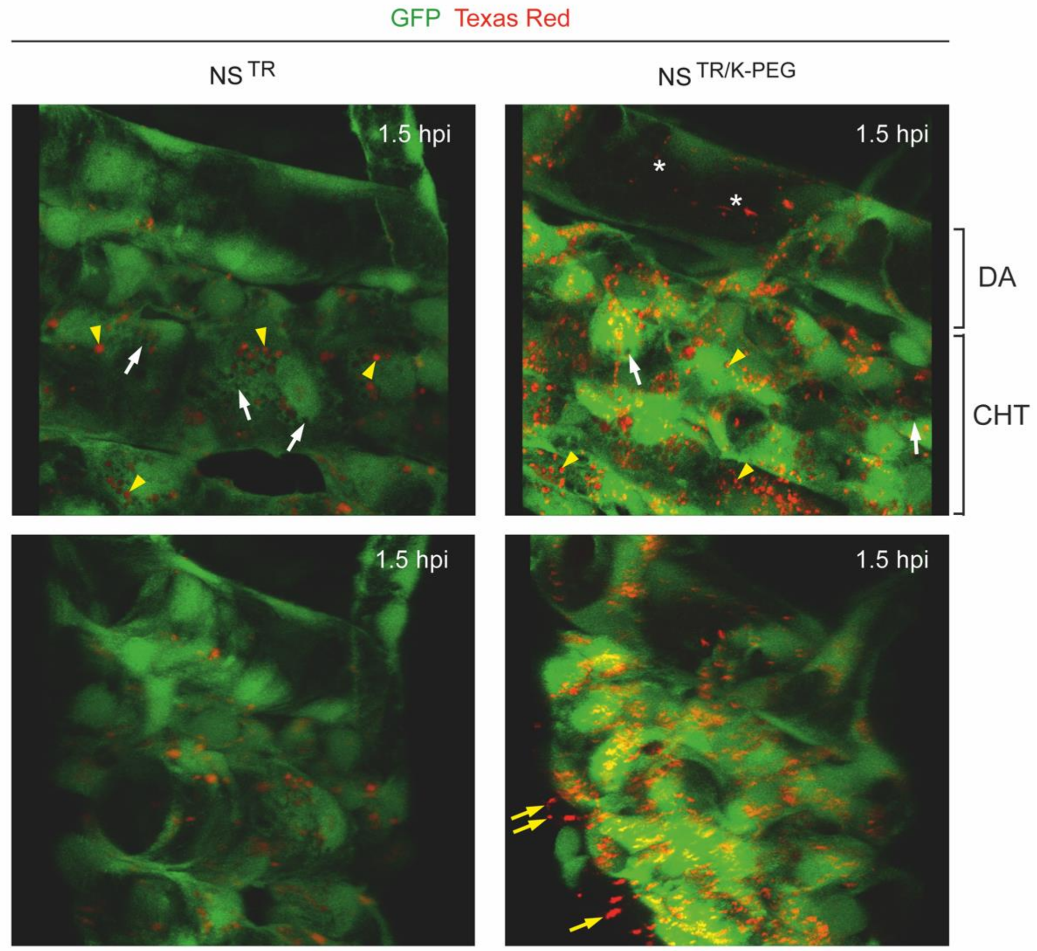

Figure S7. 3D rendering of z-stack Airyscan images of CHT and DA in *Tg(fli1: EGFP)* embryos injected with NS^{TR} or $NS^{TR/K\text{-}PEG}$ at 1.5 hpi. Vesicles in endothelial cells appear as small circular structures within the $EGFP^{+}$ cell. Vesicles with Texas Red signal (yellow arrowheads) and empty vesicles (white arrows) are observed for both NS^{TR} and $NS^{TR/K\text{-}PEG}$. Outside of the EGFP+ endothelial cells, Texas Red is either present in circulation inside $EGFP^{-}$ areas of blood vessels in the DA or in $EGFP^{-}$ cells outside of vessels. Texas Red signal both in circulation (asterisks) and interacting with EGFP- non-endothelial cells (yellow arrows) are observed for $NS^{TR/K\text{-}PEG}$. Injection of each embryo was performed as an independent experiment.

a

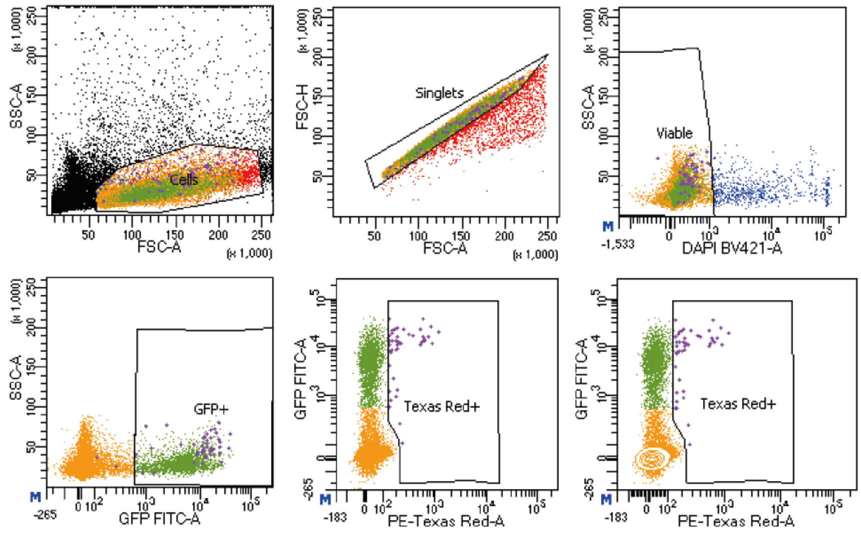

b

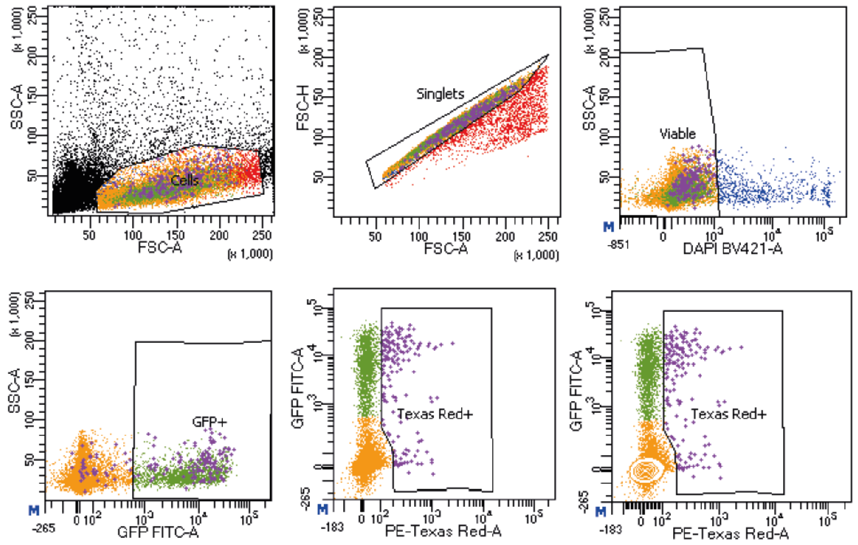

Figure S8. Isolation of Texas Red-labelled cells. FACS panels showing the strategy to isolate Texas Red-labelled cells from a cell suspension of whole zebrafish embryos injected at 2 dpf with (a) NS^{TR} or (b) $NS^{TR/K\text{-}PEG}$. Cells were initially identified by gating cells on FSC-A versus SSC-A, followed by gating on FSC-A versus FSC-H to detect singlets. Then live cells were identified by gating cells on DAPI versus SSC-A. We gated PE versus FITC-A to detect Texas Red+ cells in comparison to the GFP+ and GFP- populations, with the same relative gating threshold based on baseline values from each sample. The GFP+ cells (endothelial cells) were identified by comparing FITC-A versus SSC-A. The GFP versus Texas-Red gating plots are also shown as contour plots. Note that Texas Red+ cells were included independently of GFP-signal.

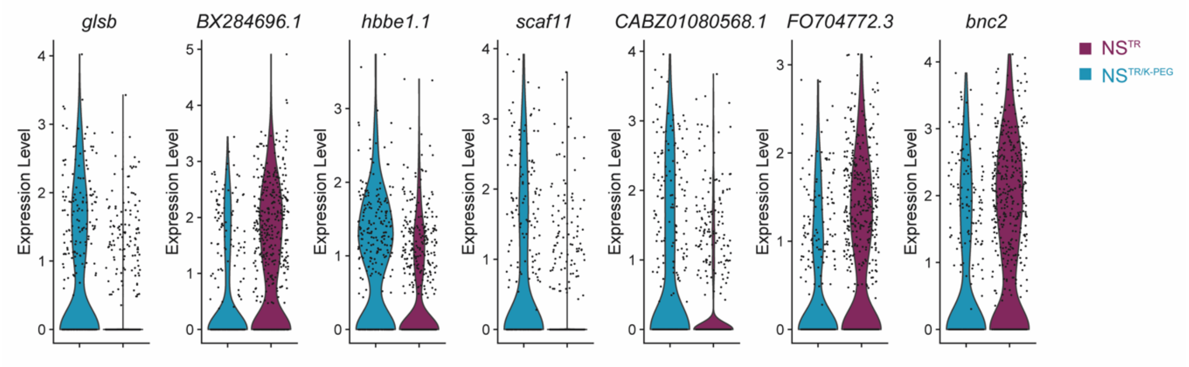

Figure S9. Differentially expressed genes of NS$^{TR/K\text{-}PEG}$ compared to NS^{TR} in scavenger/vascular endothelial cells. Differentially expressed genes in scavenger/vascular endothelial cells from embryos injected with NS$^{TR/K\text{-}PEG}$ compared to NS^{TR} were identified (FindAllMarkers) using Wilcox Rank Sum test with a logFC of 0.2 and p-value of ≤ 0.05. No significant enrichment of gene sets was detected. Note that the gene hbbe1.1 (hemoglobin beta embryonic-1.1) is only expressed in red blood cells, suggesting slight contamination of RNA from red blood cells.

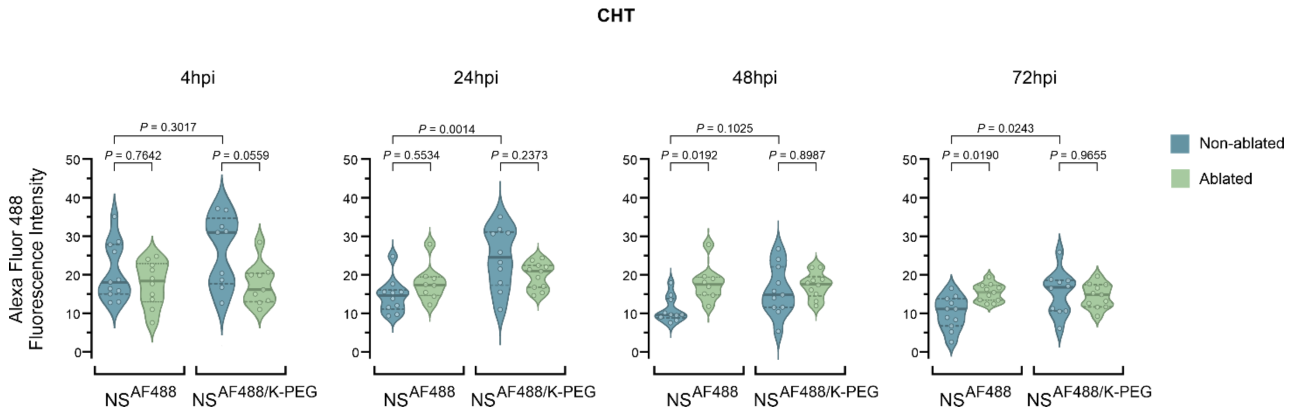

Figure S10. Effect of macrophage ablation on the fluorescence intensity at the CHT of zebrafish injected with NS^{AF488} and NS$^{AF488/K\text{-}PEG}$. Determination of NS^{TR} and NS$^{TR/K\text{-}PEG}$ levels based on confocal AF488 mean fluorescence intensity values at the CHT of MTZ- treated (ablated) and untreated (non-ablated) embryos and larvae at 4, 24, 48 and 72 hpi. Number of individually injected embryos: n=8 (24 hpi NS^{AF488} ablated, 48 hpi NS^{AF488} ablated), n=9 (4hpi NS$^{AF488/K\text{-}PEG}$ ablated, 4 hpi NS$^{AF488/K\text{-}PEG}$ non-ablated), n=10 (4hpi NS^{AF488} ablated, 24 hpi NS^{AF488} non-ablated, 24 hpi NS$^{AF488/K\text{-}PEG}$ non-ablated, 48 hpi NS^{AF488} non-ablated, 48hpi NS$^{AF488/K\text{-}PEG}$ ablated, 72 hpi NS$^{AF488/K\text{-}PEG}$ non-ablated) and n=11 (4hpi NS^{AF488} non-ablated, 24hpi NS$^{AF488/K\text{-}PEG}$ ablated, 48 hpi NS$^{AF488/K\text{-}PEG}$ non-ablated, 72 hpi NS^{AF488} ablated, 72 hpi NS^{AF488} non-ablated, 72 hpi NS$^{AF488/K\text{-}PEG}$ ablated). 72 hpi data as in Fig. 5d. *P*-values determined by one-way ANOVA followed by Tukey's multiple comparison test.

Cell type	Percentage of cells [number of cells]	
	**NS^{TR}**	**$NS^{TR/K\text{-}PEG}$**
Scavenger/Vascular Endothelial	48,14 % [506]	14,92 % [274]
Mesenchymal Neural Crest	12,84 % [135]	31,74 % [583]
Embryonic Brain	9,71 % [102]	19,98 % [367]
Red Blood Cells	6,95 % [73]	13,39 % [246]
Neuronal Neural Crest	10,28 % [108]	8,66 % [159]
Neurons	5,42 % [57]	4,19 % [77]
Pectoral Fin	3,24 % [34]	3,97 % [73]
Immune Cells	2,19 % [23]	0,93 % [17]
Musculature System	0,95 % [10]	1,63 % [30]
Skeletal Muscle	0,29 % [3]	0,6 % [11]
**Total**	**100 % [1048]**	**100 % [1837]**

Table S1. Cells interacting with NS^{TR} or $NS^{TR/K\text{-}PEG}$ detected for each cell type using single-cell RNA-sequencing. Percentage and number of cells interacting with NS^{TR} or $NS^{TR/K\text{-}PEG}$ belonging to the specified cell types. Values used for the generation of graphs presented in Figure 4e.
